# Molecular Characterization of the Hedgehog Signaling Pathway and Its Necessary Function on Larval Myogenesis in the Pacific Oyster *Crassostrea gigas*

**DOI:** 10.3389/fphys.2018.01536

**Published:** 2018-12-05

**Authors:** Huijuan Li, Qi Li, Hong Yu

**Affiliations:** ^1^Key Laboratory of Mariculture, Ministry of Education, Ocean University of China, Qingdao, China; ^2^Laboratory for Marine Fisheries Science and Food Production Processes, Qingdao National Laboratory for Marine Science and Technology, Qingdao, China

**Keywords:** hedgehog, pathway, myogenesis, cyclopamine, pacific oyster

## Abstract

Hedgehog signaling pathway participates in a chain of necessary physiological activities and dysregulation of the hedgehog signaling has been implicated in birth defects and diseases. Although substantial studies have uncovered that the hedgehog pathway is both sufficient and necessary for patterning vertebrate muscle differentiation, limited knowledge is available about its role in molluscan myogenesis. Here, the present study firstly identified and characterized the key genes (CgHh, CgPtc, CgSmo, CgGli) in the hedgehog pathway of the Pacific oyster *Crassostrea gigas*, and investigated the function of this pathway in embryonic myogenesis of *C. gigas*. Bioinformatics analysis revealed that the functional domains of the key genes were highly conserved among species. Quantitative analysis indicated that CgHh, CgPtc, CgGli mRNA began to accumulate during the blastula to gastrulation stages and accumulated throughout trochophore and into the D-shaped stage. RNA localization patterns by whole-mount *in situ* hybridization revealed that the key genes own the strongest specific staining in gastrulation, trochophore, and D-shaped stage. Hedgehog pathway genes showed a high expression level in myogenesis stage including trochophore and D-shaped stages, suggesting that the hedgehog pathway would be involved in myogenesis of *C. gigas*. In adult oysters, the key genes were expressed at various tissues, indicating that hedgehog pathway governed a series of development events. To further examine the role of hedgehog signaling in *C. gigas* myogenesis, we used cyclopamine treatment in *C. gigas* larvae to inhibit the signaling pathway. The quantification of the expression of the key genes in hedgehog pathway showed that expressions of key genes were severely down-regulated in treated larvae compared with normal larvae. The velum retractors, ventral retractors, anterior adductor, and posterior adductor muscles of larvae treated with cyclopamine at 4–6 μM for 6–12 h were severely destroyed, suggesting that the hedgehog pathway took part in the myogenesis of *C. gigas*. These findings provide a foundation for uncovering the molecular mechanisms of hedgehog signaling in molluscan physiological activity and enable us to better understand the signaling pathway involving in molluscan physiological activity.

## Introduction

Embryogenesis is a complex biological process and regulated by a mass of important cell-to-cell signaling cascades (Villavicencio et al., 2000). The principal inductive signaling pathways involved in embryogenesis are generally conserved, including TGF-β (transforming growth factor β), Wnt (Wingless and INT-1), Receptor tyrosine kinase, Notch, and hedgehog (Ingham and McMahon, 2001; Lee et al., 2001; Cornell and Eisen, 2005; Kirilly et al., 2005; Huangfu and Anderson, 2006). As one member of those cell-cell signaling pathways, hedgehog signaling is an evolutionarily conserved pathway and governs a chain of necessary embryonic development events ranging from cell proliferation, differentiation, apoptosis to morphogenesis of tissue, and organs (Weed et al., 1997). Hedgehog signaling participates in the correct development of various organs and tissue in both vertebrates and invertebrates (Heretsch et al., 2010). Furthermore, hedgehog signaling plays important roles in tissue homeostasis and dysregulation of the hedgehog pathway is relevant with developmental disorders and some diseases involving in birth defects or cancers during embryonic development (Østerlund and Kogerman, 2006).

**Figure 1:**
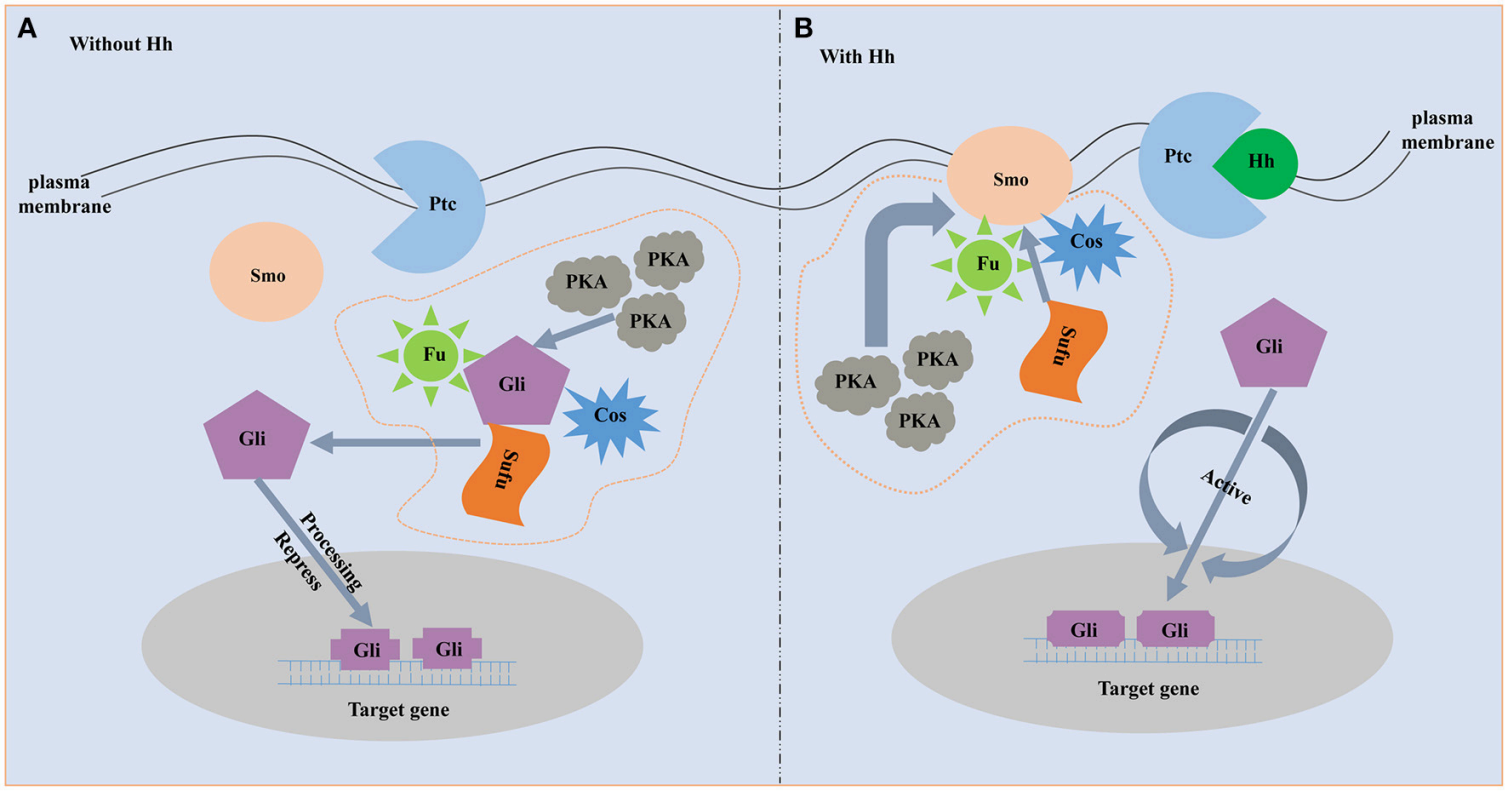
The schematic diagram of hedgehog signaling pathway. **(A)** Without Hh ligand. Fu, Su(fu) and Cos can form a complex. In the absence of Hh, the complex is able to bind to microtubules or membranes, and Gli can undergo phosphorylation by the PKA. Then the incomplete Gli functions as a transcriptional inhibitor and represses Hh target genes. **(B)** With Hh ligand. When Hh is present, binding of Hh ligand to Ptc alleviates the inhibition of Smo. Smo represses the activity of the protein complex that destroys the structure and function of the Gli. The full-length form of Gli is protected and transported into nucleus where it activates transcription.

The proteins involved in the hedgehog signaling pathway are highly conserved and include hedgehog (Hh), Patched (Ptc), Smoothened (Smo), and Gli family. Molecular characterization of the hedgehog ligands, receptors and downstream members are best understood in vertebrate and *Drosophila* (Huangfu and Anderson, 2006; Ingham and Placzek, 2006). Hh, a secreted protein, is the start of the hedgehog pathway (Villavicencio et al., 2000). Hh is firstly found in *Drosophila* to participate in embryonic segment polarity (Nüsslein-Volhard and Wieschaus, 1980). Afterward, the existence of the Hh homologs in vertebrates is proved to be a widespread phenomenon. For example, the Hh homologs are identified in mouse and chick, and they play a role in pattering of the neural tube and limb (Echelard et al., 1993; Riddle et al., 1993; Roelink et al., 1994). There are five Hh homologs in zebrafish and three of them participate in embryonic patterning (Currie and Ingham, 1996). In the mouse, there are three Hh homologs and all of them play important roles in embryonic development (Zhang et al., 2001). Hh ligand binds to the plasma membrane receptor Ptc, a 12-transmembrane protein that is a negative regulator of the pathway and highly conserved among species (Huangfu and Anderson, 2006; Østerlund and Kogerman, 2006). Ptc represses the downstream signaling when the absence of ligand, and binding of Hh ligand excuses the repression. Smo, a seven-transmembrane (TM) protein, acts downstream of Ptc and an necessary positive regulator of the hedgehog signaling (Østerlund and Kogerman, 2006). The seven-TM region structure of Smo is relatively conserved among species and strongly resembles the Frizzled protein family (Nusse, 2003). Gli is the downstream transcription factor and the end of the pathway. Active Smo regulates the bifunctional transcription factor Gli. Full-length form Gli protein is a transcriptional activator while broken or incomplete Gli protein becomes a transcriptional repressor and blocks the activity of hedgehog signaling (Matus et al., 2008). During the absence of Hh ligand, Ptc as an inhibitor blocks the ability of Smo to activate the hedgehog signaling (Figure 1). On this occasion, the downstream transcription factor Gli is phosphorylated and processed by an intracellular protein complex including PKA, Slimb, GSK3β and Fused. Then the incomplete Gli functions as a transcriptional inhibitor and represses the activity of the hedgehog signaling pathway (Jia et al., 2002; Price and Kalderon, 2002). When Hh is present, binding of Hh ligand to Ptc alleviates the inhibition of Smo (Figure 1). Subsequently, Smo represses the activity of the protein complex that destroys the structure and function of the Gli. The full-length form of Gli is protected and transported into nucleus where it activates transcription (Méthot and Basler, 2000).

Hedgehog signaling has been defined as an important regulator of myogenesis in vertebrates. Hedgehog signaling determines the fast and slow muscle fate and affects the muscle types in zebrafish embryo (Currie and Ingham, 1996; Blagden et al., 1997; Du et al., 1997). Overexpression of Hh in zebrafish is able to convert the entire myotome into slow muscle fibers (Blagden et al., 1997; Du et al., 1997). Hedgehog signaling also has a later effect on regulating the differentiation of the dermomyotome in zebrafish. Hedgehog signaling has no roles in the initial formation of the dermomyotome but is related with its differentiation into fast muscle fibers (Feng et al., 2006). Hh governs the proliferation and differentiation of myoblasts during chick limb muscle and mouse hypaxial muscle development (Duprez et al., 1998; Kruger et al., 2001). In mice, Myod is a myogenic regulatory factor that is in charge of the survival and proliferation of myoblasts (Braun et al., 1992). Pax 3 has a role in development of the somatic mesoderm muscles (Goulding et al., 1994). Hedgehog signaling has a role in expression of the Pax-3 and MyoD in mouse (Duprez et al., 1998). The ectopic expression of Hh results in the upregulation of myogenic regulatory factors (MRFs) in mice hypaxial musculature.

Though there are abundant data showing that hedgehog signaling is related with the vertebrate muscle differentiation, knowledge on the role of this pathway in invertebrate myogenesis is little (Grimaldi et al., 2008). A Hh-related gene was discovered to participate in the normal development of obliquely striated muscles in leech proboscis (Kang et al., 2003). In Mollusca, the role of hedgehog signaling in myogenesis is investigated only in *Sepia officinalis*. The hedgehog signaling is involved in correct patterning of obliquely striated muscle fibers in cuttlefish mantle (Grimaldi et al., 2008).

The Pacific oyster *Crassostea gigas* is the most widely cultivated marine bivalves and widely distributed throughout the world (Zhu et al., 2016). *C. gigas* is also becoming an interesting model species in the developmental biology because it owns the typical molluscan development stages, including planktonic laravae and metamorphosis. Though *C. gigas* possess highly economic and scientific value, knowledge about the molecular mechanisms of myogenesis is still obscure up to now. Here, we choose the *C. gigas* as a bivalve animal model to further study the conserved role of Hedgehog signaling in myogenesis. We obtained the full length cDNA of *C. gigas* Hh, Ptc, Smo, Gli (CgHh, CgPtc, CgSmo, CgGli). Subsequently, a detailed bioinformatics analysis on the molecular characterization of the hedgehog pathway genes was conducted. The spatial and temporal expression pattern of the hedgehog signaling members was studied using RT-qPCR and whole-mount *in situ* hybridization methods. To reveal the function on *C. gigas* myogenesis of hedgehog signaling, we inhibited the signaling with cyclopamine treatment in *C.gigas* embryo. Then the expression profiles of target genes and F-actin were analyzed by mean of RT-qPCR and fluorescent phalloidin staining respectively. The study was not only the first to reveal the molecular characterization and expression files of hedgehog signaling primary genes in bivalva, but also the first to investigate its function on bivalve myogenesis. The results would provide a foundation for analyzing the molecular mechanisms of hedgehog signaling in invertebrate and enable us to better understand the function of hedgehog signaling in invertebrate myogenesis.

## Materials and Methods

### Samples Collection

Adult *C.gigas* were collected from an oyster farm in Laizhou in Shandong province, China. Adult tissue including gonad, hemolymph, mantle, striated, and smooth adductor muscles, gill, labial palp, digestive gland were frozen in liquid nitrogen and then stored at −80°C until further processed. Larval culture was executed as Wang et al. described (Wang et al., 2012). Embryo and larvae were sampled at the following stages: fertilized eggs, two-cell stage, four-cell stage, blastula stage, gastrula stage, trochophore, D-shaped larvae, unbo larvae and eyed larvae. Samples stored at RNA store (Dongsheng Biotech, China) at −20°C were used for extraction of RNA. For whole-mount *in situ* hybridization, samples were fixed in 4% paraformaldehyde (PFA) in 0.1 M phosphate buffered saline (PBS, pH = 7.3) for 2–3 h at 4°C. Afterwards, the larvae were rinsed three times for 10 min in methanol and then were stored at −20 °C in methanol. Samples including D-shaped larvae, umbo larvae and eyed larvae were anesthetized with 7.5% MgCl_2_ prior to fixation. Samples used for fluorescent phalloidin staining were fixed in 4% PFA in 0.1 M PBS for 2–3 h at room temperature (RT). Afterwards, the samples were rinsed three times for 10 min in 0.1 M PBS at RT and then were stored at 4°C in 0.1 M PBS with 0.1% NaN_3._

### Total RNA Isolation and Synthesis of cDNA

TRIzol reagent (Invitrogen, USA) was used to extract the total RNA from *C.gigas* samples following the manufacturer's protocol. Agarose gel (1%) electrophoresis with 10 × loading buffer (Takara) was used to detect the quality of RNA. The RNA concentration and purity were verified at optical density (OD)260/(OD)280 with a NanoDrop 2000 (Thermo Scientific**)** spectrophotometer. PrimeScriptTM Reverse Transcription Kit (Takara) was used to synthesize first-strand cDNA according to the manufacturer's instructions. The cDNA was preserved at −20°C.

### Cloning the Full-Length cDNA of Hedgehog Signaling Genes

The internal fragment of hedgehog genes were amplified by PCR according to the bioinformatics prediction of the gene models (Hh: LOC105317164; Smo: LOC105318081; Ptc: LOC105326154; Gli: LOC105322770). The template was the cDNA reverse translated from RNA of *C.gigas* adductors. Specific primers were designed by the Primer Premier 5.0 software (Premier Biosoft International, Palo Alto, CA) based on the predicted sequence. The thermocycling program of the PCR was performed as follows: pre-denaturation at 94°C for 3 min, 94°C for 30 s, Tm for 30 s, 72°C for 45 s, 35 cycles; 72°C for 5 min. The PCR products were detected by 1.5% agarose gel electrophoresis and then sequenced using an ABI 3730 Genetic Analyzer (Applied Biosystems).

According to the obtained internal fragment sequence, the specific primers for 3′ and 5′ RACE reactions were designed. The 3′ and 5′ terminal cDNA were cloned using the RACE method with the SMARTer® RACE 5′/3′ Kit (Clontech). The 3′ and 5′ RACE PCR reactions were amplified with Tks Gflex™ DNA Polymerase (Takara) according to the following conditions: 98°C for 1 min; 98°C for 10 s, Tm for 10 s, 68°C for 20 s, 35 cycles. The PCR products were purified using DNA purified kit (Tiangen, China) and ligated into the pEASY-simple Blunt vector (Trans Biotech, China) and sequenced in both directions (Sangon Biotech, China).

### Molecular Characteristic and Phylogenetic Analysis

The 3′, 5′ terminal sequence and internal fragment were assembled using SeqMan (DNAStar) software to acquire the full length cDNA of hedgehog signaling genes. The DNAMAN (Lynnon Biosoft) software was used to predict the open reading frame and translate the cDNA sequence to the protein sequence. The isoelectric point (pI) and molecular weight (MW) of the deduced amino acid sequences were predicted using the Compute pI/MW Tool at the ExPAsy site (http://web.expasy.org/compute_pi/). The TMHMM server v.2.0 (http://www.cbs.dtu.dk/services/TMHMM-2.0/) was used to predict transmembrane helices. The NetPhos server (http://www.cbs.dtu.dk/services/NetPhos) was used to generate neural network predictions for phosphorylation sites. The ScanProsite program (https://prosite.expasy.org/scanprosite/) was used to analyze the conserved motif. The PredictProtein program (https://ppopen.informatik.tu-muenchen.de/) was used to predict the secondary structure and transmembrane helices of protein. The signal peptide and three-dimensional structure of the protein were analyzed with the SignalP 4.1 (http://www.cbs.dtu.dk/services/SignalP/) and PHYRE2 servicer (http://www.sbg.bio.ic.ac.uk/phyre2). The ClustalW (Lynnon Biosoft, Los Angeles, CA) was used to align the protein sequences and modified by the ESPript 3.0 (Robert and Gouet, 2014). A phylogenetic tree was constructed by the Neighbor-Joining analysis with 1,000 bootstrap replicates using MEGA 7.0 software (Kumar et al., 2016). Protein sequence logos were generated using the Java application LogoBar (Pérez-Bercoff et al., 2005).

### Expression Analysis of Key Genes by Real-Time Quantitative PCR

The qRT-PCR primers for the hedgehog signaling genes were designed, and their specificities were detected by conventional PCR and melting curve analyses. Ribosomal protein S18 (RS18) and elongation factor 1-α (EF1-α) were used as the internal control in the larvae and adult samples respectively (Du et al., 2013). cDNAs of hedgehog signaling genes were amplified using SYBR® Premix Ex Taq™ II kit (Takara) in a LightCycler® 480 real-time PCR system (Roche) according to the manufacture's protocols. The 10 μL qRT-PCR reaction contained 5 μL 2 × SYBR Premix ExTaq (Takara), 1.8 μL of each primer, and 1 μL diluted cDNA. The RT-qPCR amplification was conducted at 95°C for 30 s, followed by 40 cycles at 95°C for 5 s, 60°C for 20 s and 72°C for 20 s. The relative expression was calculated by the 2^−ΔΔCt^ method (Livak and Schmittgen, 2001). All data were given in the light of relative mRNA expression levels as means ± SE (*n* = 6). The IBM SPSS Statistics 22 was used to analyze the significant differences between the means. All the data analyses were performed using one-way ANOVA followed by a multiple comparison. Differences were considered statistically significant at *P* < 0.05.

### Whole-Mount *in situ* Hybridization

Whole mount *in situ* hybridization was performed using digoxigenin-labeled sense and anti-sense probes synthesized following SP6- and T7-mediated *in vitro* transcription (MEGAscript kit, Ambion) using a DIG-RNA labeling Kit (Roche) from the clone of cDNAs fragment of target genes. The experiment was conducted using the protocol described by Thisse and Thisse (2008) with some adjustments. Stored samples were rehydrate stepwise into PBST for 30 min, and then prehybridized with hybridization buffer (50% formamide, 50 μg/ml of heparin, 5 × SSC, 500 μg/ml tRNA, 9.2 mM citric acid, 0.1% Tween-20). Sense or anti-sense probes concentrations was 1.0 to 2.0 ng/μl and hybridizations were performed at 65°C overnight. Unbound probes were rinsed by a series of low-salt (2 × SSC, 0.2 × SSC) and samples were incubated in blocking solution for 2–4 h. embryo and larvae were incubated in anti-digoxigenin antibody (Roche) at 1:5,000 in blocking solution overnight at 4°C. Samples were then washed several times in MABT (150 mM sodium chloride, 100 mM maleic acid, 0.1% Tween-20, pH 7.5), and in alkaline Tris buffer for 3 × 5 min. Color reactions were performed with 2% NBT/BCIP solution for 2 h in at room temperature or overnight at 4°C. Specimens were photographed on a fluorescence light microscope (Olympus BX53) with digital camera (Olympus DP73).

### Cyclopamine Treatment

Cyclopamine, a known blocker of hedgehog signaling pathway from invertebrates and vertebrates (Incardona et al., 2000; Kang et al., 2003), was diluted to a final concentration of 0.5, 1, 2, 4, 6 μM in sea water (from a stock solution 10 Mm in ethanol). Embryos were selected for treatments when they developed into trochophore stage. Experimental embryos (trochophore stage) were cultured in cyclopamine for 12 h (up to D-shaped stage). Control embryos were cultured in sea water with 0.1% ethanol. Treatments were done in 12 L bucket with 10 L of filtered fresh seawater. The rearing conditions retained same for control and experiment embryos. Water temperature was maintained at 23–24°C, with salinity at 30 psu and densities at 10 embryo/ml. Samples were collected and fixed for RNA extraction and F-actin staining, as described above.

### F-Actin Staining and Confocal Microscopy

The specimens were permeabilized in PBS containing 2% Triton-X 100 (PBT, pH = 7.3) overnight at room temperature. Actin staining was conducted by fluorescence labeling of filamentous F-actin with Phalloidin-iFluor™ 488 Conjugate (AAT Bioquest) in a 1:1,000 dilution in PBT for 24 h in the dark. Then the larvae were washed three times for 10 min each and mounted in Fluoromount G (Southern Biotech) between two coverslips to allow scanning from both sides. Acquisition of confocal images were performed on a Nikon ECLIPSE Ti confocal laser scanning microscope equipped with the software NIS-Elements (Version 4.0). Optimization and adjustment of brightness and contrast was conducted with Image J software (National Institutes of Health) and Photoshop CS5 (Adobe).

## Results

### cDNA Cloning and Sequence Analysis of Key Hedgehog Pathway Genes

The full-length cDNA fragment of CgHh was 3,676 bp, containing a 5′-untranslated region (5′-UTR) of 1,176 bp, a 3′-untranslated region (3′-UTR) of 1,276 bp, and a putative open reading frame (ORF) of 1,224 bp encoding a 407-amino acid protein with an ATG start codon and TGA stop codon. A polyadenylation signal, AATAAA, was found 12 bases upstream from the poly (A) tail. The nucleotide and deduced amino acid sequences of Hh were shown in Figure 1. According to the amino acid sequence, the predicted molecular mass and the isoelectric point of CgHh were 44.99 kDa and 7.67, respectively. The total number of positivity (Arg + Lys) and negativity (Asp + Glu) in the amino acid composition of CgHh was 44 and 43, respectively. A signal petide was predicted by the SignalP 4.1 Server: the signal peptide cleavage sites of CgHh were Ala16 and Phe17, suggesting that it was a secretory protein. The CgHh protein contained HH-signal, Hint-N, and Hint-C conserved domains predicted by SMART software. The HH-signal domain is the N-terminal domain of CgHh protein, which is responsible for signaling following cleavage from the C-terminal domain. The Hint-N and Hint-C represented the C-terminal and intein domain of CgHh protein. The Hint domain was split to accommodate large insertions of endonucleases. A conserved intein N-terminal splicing motif were detected in the Hint-N domain (Figures S1, S5). The putative protein secondary structure of CgHh contained eight alpha helices and twenty one beta strand structures. The tertiary structure of Hh protein was based on template c3m1nB, which shared 78% identity with CgHh protein.

The full length CgPtc cDNA was 5,266 bp, including a 288 bp 5′-UTR, a 3,957 bp ORF encoding a 1,318 amino acid protein with predicted molecular weight of 146.51 kDa and a pI of 6.46 and a 1,021 bp 3′-UTR with a typical polyadenylation signal sequence AATAAA and a poly (A) tail. CgPtc was not a secreted protein for the reason that no signal peptide was detected in the Ptc protein according to SignalP 4.1 analysis with the D-score of 0.105 (cutoff score of 0.450). CgPtc protein included a Sterol-sensing domain and a Patched domain at the position 433–587 and 908–1,155 respectly. CgPtc protein owned one conserved Sterol-sensing domain (SSD) motif (Figures S2, S6). CgPtc is a protein with 12 transmembrane domains and its transmembrane helix region were at located among amino acid positions 56–78, 407–429, 442–464, 468–490, 518–540, 550–572, 724–746, 1002–1024, 1026–1048, 1058–1080, 1101–1123, 1133–1155. The secondary structure prediction showed that 55% of the total amino acids were in alpha helix and 5% formed beta strand. There were 27 Serine, 4 Tyrosine and 8 Threonine phosphorylation sites by protein kinase A (PKA) and casein kinase I (CKI).

The full length of cDNA CgSmo contained a 5′-UTR of 387 bp and a 3′-UTR of 802 bp with a poly (A) tail (**Figure 3**). The ORF of Smo was 3,021 bp encoding 1,006 amino acids with predicted theoretical isoelectric point of 9.51 and molecular weight of 112.3 kDa. The CgSmo was a secretory protein and the signal pepide cleavage sites of CgSmo were Ser21 and Thr22. Smo protein contained the FRI and Frizzled conserved functional domains. Seven transmembrane helix region detected by the TMHMM program were at located among amino acid positions 204–226, 233–255, 290–312, 333–355, 375–397, 426–448, 490–512, 683–700 (Figure S3). The predicted phosphorylated sites of CgSmo were mainly located in the C-terminal tail (Figures S3, S7). There were 27 Serine, 8 Tyrosine and 4 Threonine phosphorylation sites by protein kinase A (PKA) and casein kinase I (CKI). Two conserved motifs were found in accordance with CgSmo amino acids, including a Fruzzled (FZ) and G-protein coupled receptors family motifs at the positions of 26–150 and 202–467 respectively (Figure S3).

The cDNA of CgGli was 5,278 bp, containing 402 bp 5′-UTR, 4,749 bp ORF and 127 bp 3′-UTR. The ORF of 4,749 bp was predicted to encode a 1,582 amino acid. The estimated molecular weight and pI were 176.37 kDa and 7.84 respectively. There was no signal peptide and transmembrane domains in CgGli protein. The CgGli protein included five conserved ZnF_C2H2 functional domains. The zinc finger domains were relatively small protein motifs which contained multiple finger-liker protrusions that made tandem contacts with their target molecule. There were four zinc finger C2H2 type motifs in CgGli protein (Figures S4, S8). The tertiary structure of CgGli protein was based on template c2gliA, which shared 81% identity with CgGli protein.

### Homology and Phylogenetic Analysis of Key Hedgehog Pathway Genes

**Figure 2:**
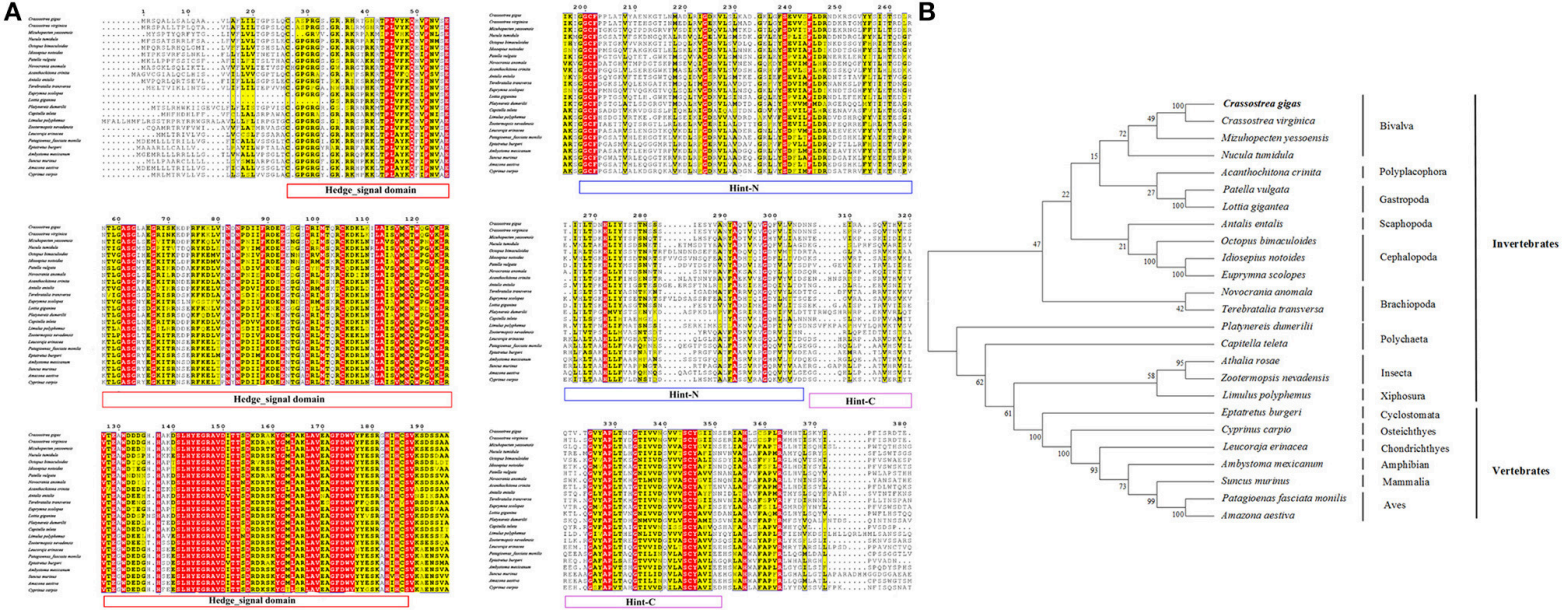
**(A)** Multiple sequence alignment of the CgHh amino acid sequence among species. The conserved functional structure are marked in box. Identical residues are shown as white letters with red background, and similar residues are shown as black letters with yellow background. **(B)** Neighbor-joining phylogenetic tree based on the amino acid sequences of the CgHh.

The amino acid sequence alignment of CgHh with Hh from other vertebrate and invertebrate species showed a moderate degree of sequence similarity. It exhibited high similarity to Hh of the genus *Crassostrea* such as *Crassostrea virginica* (91%). A high sequence similarity was detected in other Bivalva species such as *Mizuhopecten yessoensis* (59%), *Nucula tumidula* (57%). In Mollusk, A high similarity was founded in Gastropoda, Cephalopoda, Scaphopoda, Polyplacophora, including *Patella vulgate* (55%), *Lottia gigantean* (57%); *Idiosepius notoides* (55%), *Euprymna scolopes* (53%), *Octopus bimaculoides* (53%); *Antalis entails* (50%); *Acanthochitona crinite* (55%). Furthermore, A moderate similarity was analyzed in other invertebrates: Annelida (50–51%), Insecta (47–52%); vertebrates: Osteichthyes (48%), Chondrichthyes (50%), Aves (49–50%), Mammalia (48%). In addition, the Hedge_signal domain and Hint-C partial domain were evolutionally conserved among the vertebrates and invertebrates (Figure 2A). The phylogenetic analysis of Shh showed that the *Crassostrea* species was clustered together, and was closely related to the *M. yessoensis* (Figure 2B). All the Mollusca species were clustered together and formed two branches. The Bivalva, Polyplacophora and Gastropoda were clustered together, the Scaphopoda and Cephalopoda were clustered together as another branch.

**Figure 3:**
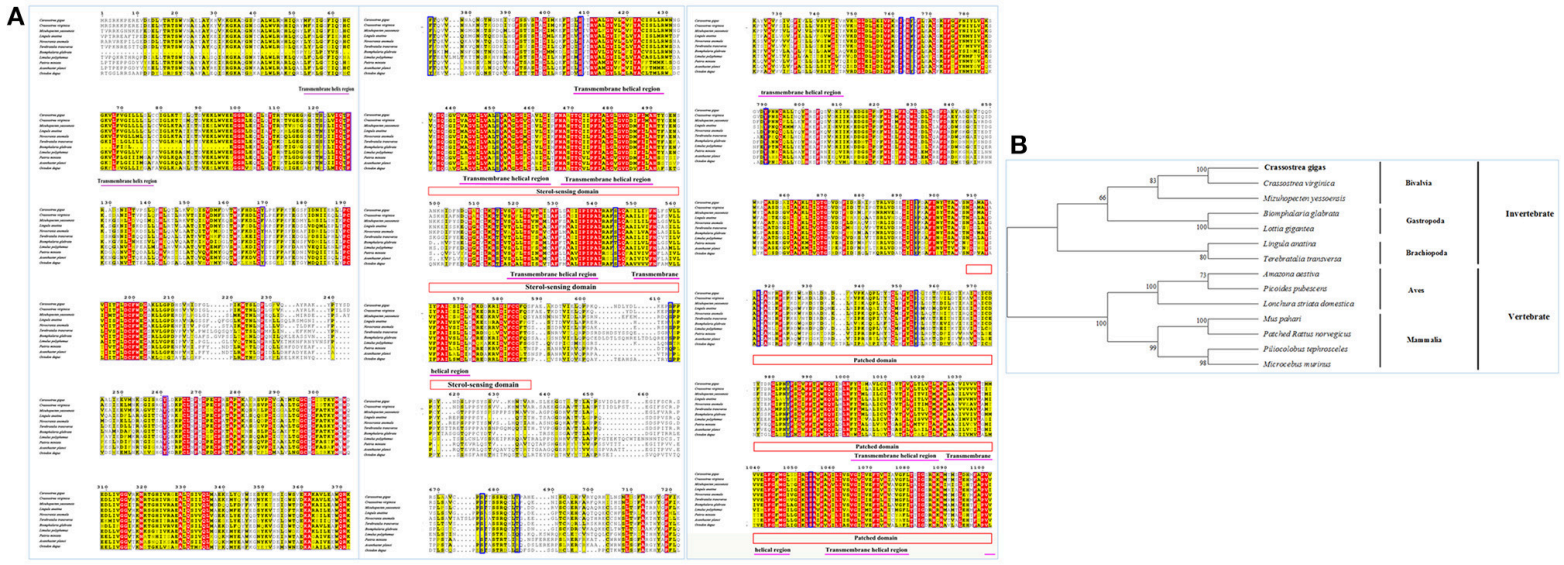
**(A)** Multiple sequence alignment of the CgSmo amino acid sequence among species. The conserved functional structure are marked in box. Identical residues are shown as white letters with red background, and similar residues are shown as black letters with yellow background. **(B)** Neighbor-joining phylogenetic tree based on the amino acid sequences of the CgSmo.

Sequence alignment of CgPtc with Ptc from other species showed that it displayed an extremely high similarity to Ptc of the *C. virginica* (89%; Figure 3A). A moderate degree of amino acid sequence similarity was found in other mollusks such as *M. yessoensis* (63%), *Terebratalia transversa* (55%), and *Biomphalaria glabrata* (56%). It also exhibited moderate similarity to Ptc form other species: *Novocrania anomala* (59%), *Lingula anatine* (55%), *Limulus Polyphemus* (50%), *Patiria miniata* (48%), *Octodon degus* (46%) and *Acanthaster planci* (45%). In addition, the transmembrane helical region, sterol-sensing domain and patched domain were evolutionally conserved among vertebrates and invertebrate species. A majority of phosphorylation sites were highly conserved located in the position of 121, 169, 261, 373, 409, 451, 515, 545, 613, 676, 685, 894, 916, 954, 984, 1,054. The phylogenetic analysis showed that all the invertebrates were clustered together and formed three branches: Bivalvia, Gastropoda and Brachiopoda. *C. gigas, C. virginica*, and *M.yessoensis* were clustered together. *B.glabrata* and *L.anatina* were clustered together as another branch. The vertebrates were clustered together and formed two branches including Aves and Mammalia (Figure 3B).

**Figure 4:**
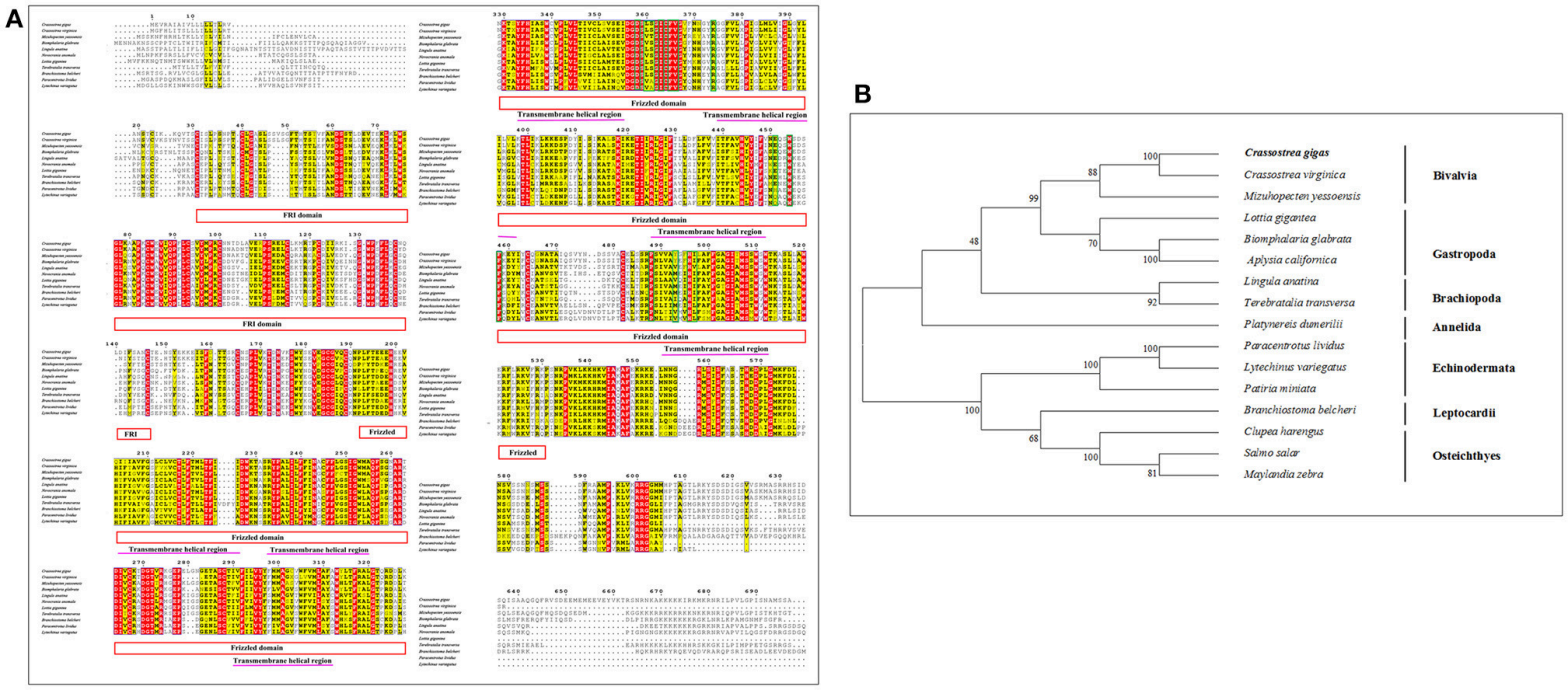
**(A)** Multiple sequence alignment of the CgPtc amino acid sequence among species. The conserved functional structure are marked in box. Identical residues are shown as white letters with red background, and similar residues are shown as black letters with yellow background. **(B)** Neighbor-joining phylogenetic tree based on the amino acid sequences of the CgPtc.

Multiple sequence alignment of Smo showed that CgSmo had the highest identity with *C.virginica* at 87% in the *Crassostrea* species (Figure 4A). A moderate degree of sequence similarity was found in mollusks including *M.yessoensis* (53%), *B.glabrata* (49%), *L.anatina* (49%). The transmembrane helical region, FRI domain and Frizzled domain were highly conserved among invertebrates and vertebrates. The ligand binding sites at the position of 374, 451, 454, 458, 488, 493, 497 were absolutely conserved among species. According to deduced amino acid sequences of CgSmo from other species, a neighbor–joining phylogenetic tree was formed to identify the evolutionary position of CgSmo. In general, species from the same class were clustered together and developed into a separated subgroup. All the Mollusca species were clustered together and formed two branches including Bivalvia and Gastropoda; Brachiopoda species including *L.anatina* and *T. transversa* were clustered together as another branch (Figure 4B). In general, the phylogenetic results were consistent with traditional taxonomy.

**Figure 5:**
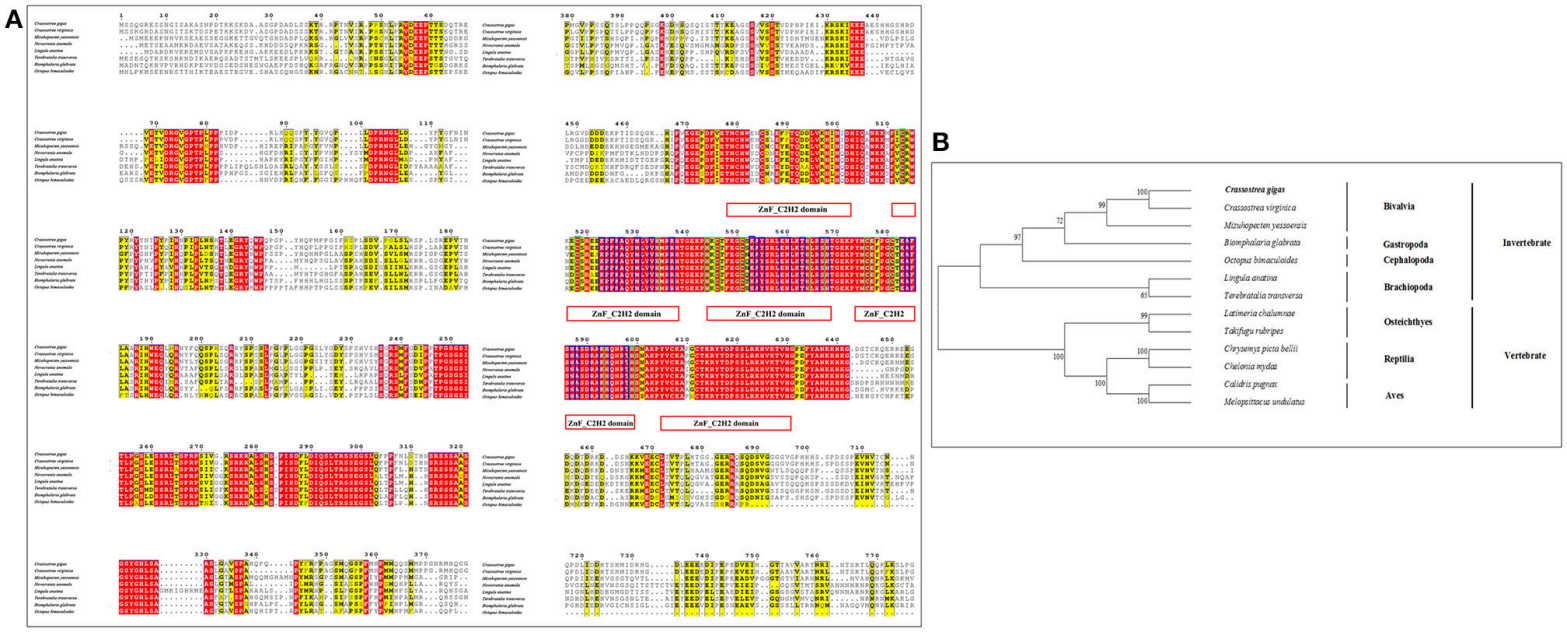
**(A)** Multiple sequence alignment of the CgGli amino acid sequence among species. The conserved functional structure are marked in box. Identical residues are shown as white letters with red background, and similar residues are shown as black letters with yellow background. **(B)** Neighbor-joining phylogenetic tree based on the amino acid sequences of the CgGli.

Similar to CgHh, CgPtc and CgSmo, CgGli showed a high similarity to *C. virginica* (91%) and a moderate degree identity among other mollusks including *M. yessoensis* (55%), *O. bimaculoides* (62%), *B. glabrata* (55%). For *Brachiopoda*, CgGli exhibited a moderate similarity such as *L.anatina* (55%) and T. transversa (44%; Figure 5A). The putative nucleic acid binding sites at the position 555, 558, 559, 563, 567, 568, 583, 585, 586, 589, 590, 593, 594, 598 were highly conserved. The Zn binding sites owning a C-C-H-H conserved feature residue pattern and five ZnF_C2H2 domain showed a high identity among species. All the invertebrates were clustered together and came into being four groups including Bivalvia, Gastropoda, Cephalopoda and Brachiopoda. In Bivalvia, *Crassostrea* species were clustered together and had a closer relationship with *M.yessoensis*. Three branches including Osteichthyes, Reptilia and Aves were established in vertebrates (Figure 5B).

### Temporal Expression Patterns of Key Hedgehog Pathway Genes

**Figure 6:**
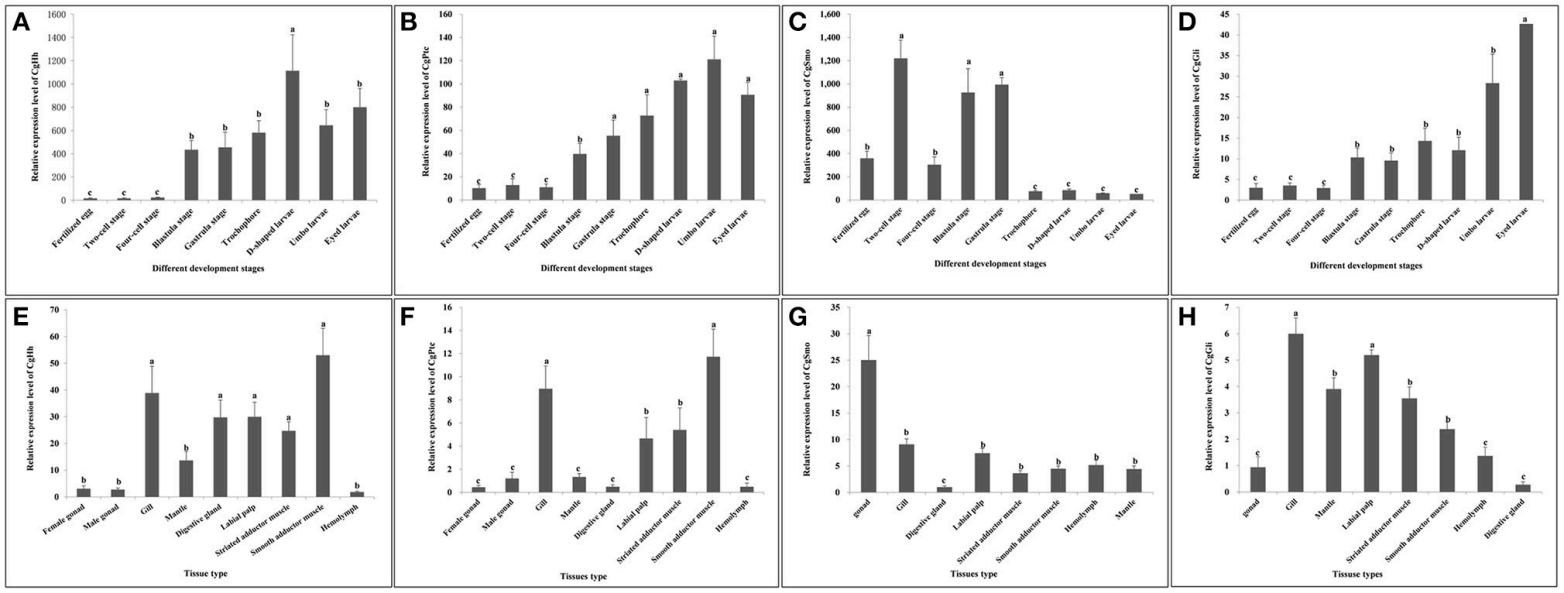
**(A–D)** Expression profiles of key genes during embryo-larval developmental stages. **(E–H)** Expression profiles of key genes in adult different tissues. Different letters indicated significantly different (*P* < 0.05). The letters “a,” “b,” and “c” indicate the significance of the differences between two groups. Firstly, the whole mean of gene expression is ranked from large to small. The maximum average is marked the letter “a” and this average is compared with the following averages. Where the difference is not significant, the letter “a” is marked; otherwise, the letter “b” is marked. Secondly, the maximum average marked “b” is used as the standard deduction and compared with the following unmarked averages. Where the difference is not significant, the letter “b” is marked; otherwise, the letter “c” is marked.

The temporal expression patterns among different embryo-larval developmental stages and different adult tissues of several key hedgehog pathway genes were examined by RT- qPCR (Figure 6). CgHh mRNA was not detected until blastula stage (*P* < 0.05) during the embryo stages. CgHh mRNA began to accumulate during blastula stage to trochophore stage and peaked in D-shaped stage (*P* < 0.05). During umbo larvae and eyed larvae, CgHh gene expression showed a sharply decrease (*P* < 0.05). The CgHh mRNA expression levels in different tissues including the female gonad, male gonad, gill, mantle, digestive gland, labial palp, striated adductor muscle, smooth adductor muscle, and hemolymph were investigated. The highest expression level was detected in smooth adductor muscle (*P* < 0.05), followed by gill, digestive gland, labial palp and striated adductor muscle. Relatively low expression were discovered in mantle, gonad and hemolymph (*P* < 0.05). CgPtc mRNA expression was relatively low during fertilized egg and four-cell stage until development into blastula stage (*P* < 0.05). CgPtc gene expression showed an overall increase pattern from blastula stage through gastrula stage and trochophore stage into D-shaped stage. A small decreased expression was observed in umbo larvae. Similar to CgHh, CgPtc mRNA expression showed a highest level in the smooth adductor muscle and a higher level in the gill and striated adductor muscle. A lower expression was detected in the labial palp, mantle, gonad, digestive gland and hemdymph (*P* < 0.05). CgSmo mRNA expression began fertilized egg and increased throughout the blastula stages and gastrula stage. A sharply decrease expression was observed from trochophore stage to eyed larvae (*P* < 0.05). CgSmo mRNA was expressed in a variety of tissues and had a highest expression in gonad tissue. A lower expression was observed in hemolymph, gill, labial palp and adductor muscles. CgGli mRNA was expressed in a similar pattern to CgHh. There was an overall increasing trend from the blastula stage through trochophore stage and a large increase at the eyed larvae stage. The CgGli gene was expressed in all tested tissues and the highest expression level was founded in gill. Relatively lower expression levels were detected in labial palp, gill, mantle, and adductor muscle.

### RNA Localization Patterns of Key Hedgehog Pathway Genes

**Figure 7:**
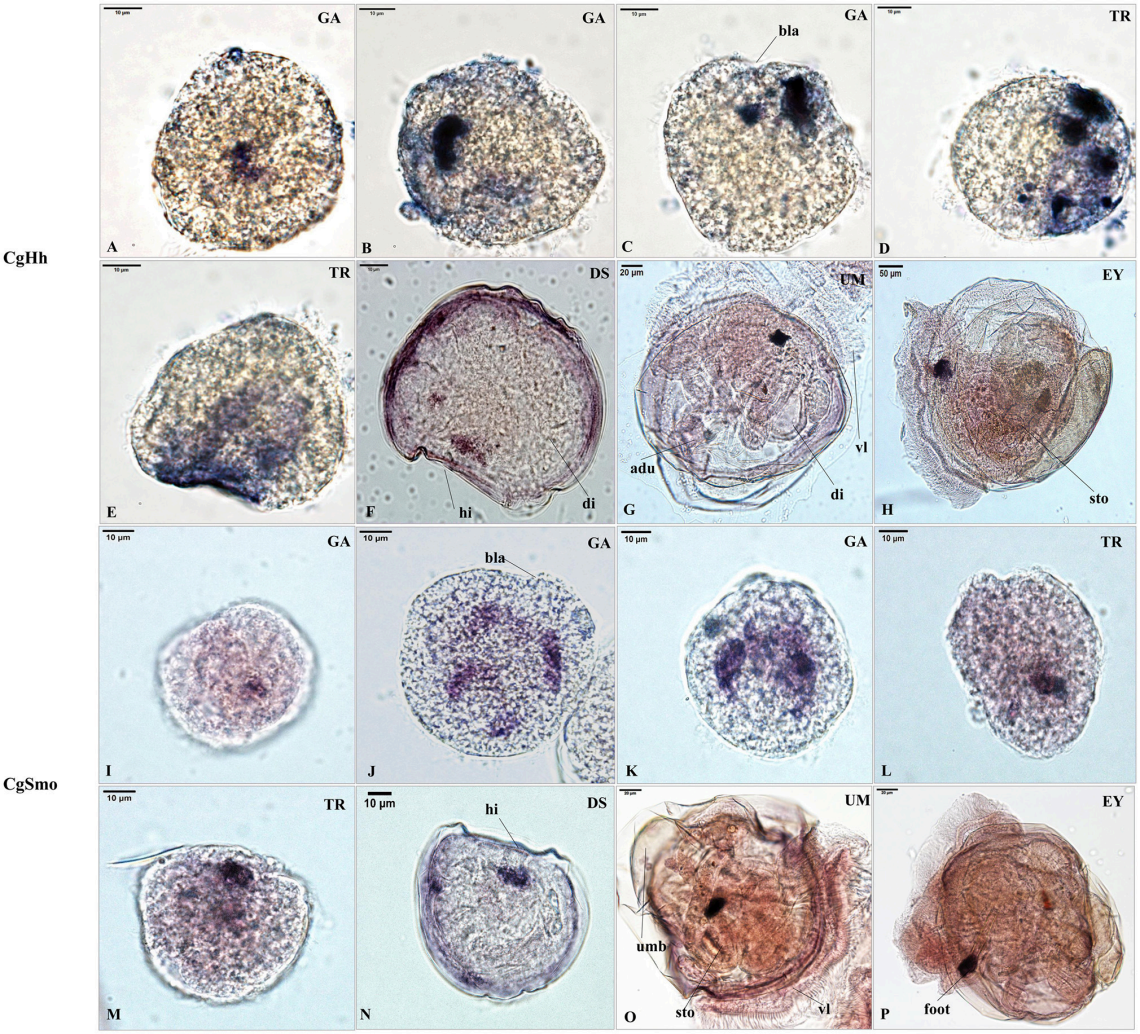
RNA localization patterns of key hedgehog pathway genes during embryonic to larval stage. **(A–C)** RNA localization patterns of CgHh during gastrula stages. **(D,E)** RNA localization patterns of CgHh during trochopore stages. **(F)** RNA localization patterns of CgHh during D-shaped stages. **(G)** RNA localization patterns of CgHh during umbo stages. **(H)** RNA localization patterns of CgHh during eyed stages. **(I–K)** RNA localization patterns of CgSmo during gastrula stages. **(L,M)** RNA localization patterns of CgSmo during trochopore stages. **(N)** RNA localization patterns of CgSmo during D-shaped stages. **(O)** RNA localization patterns of CgSmo during umbo stages. **(P)** RNA localization patterns of CgSmo during eyed stages. GA, gastrulae stage; TR, trochophore stage; DS, D-shaped stage; UM, umbo larvae stage; EY, eyed-larvae stage; bla, blastopore; hi, hinge; di, digest gland; vl, vlum; um, umbo; fo, foot; adu, adductor.

**Figure 8:**
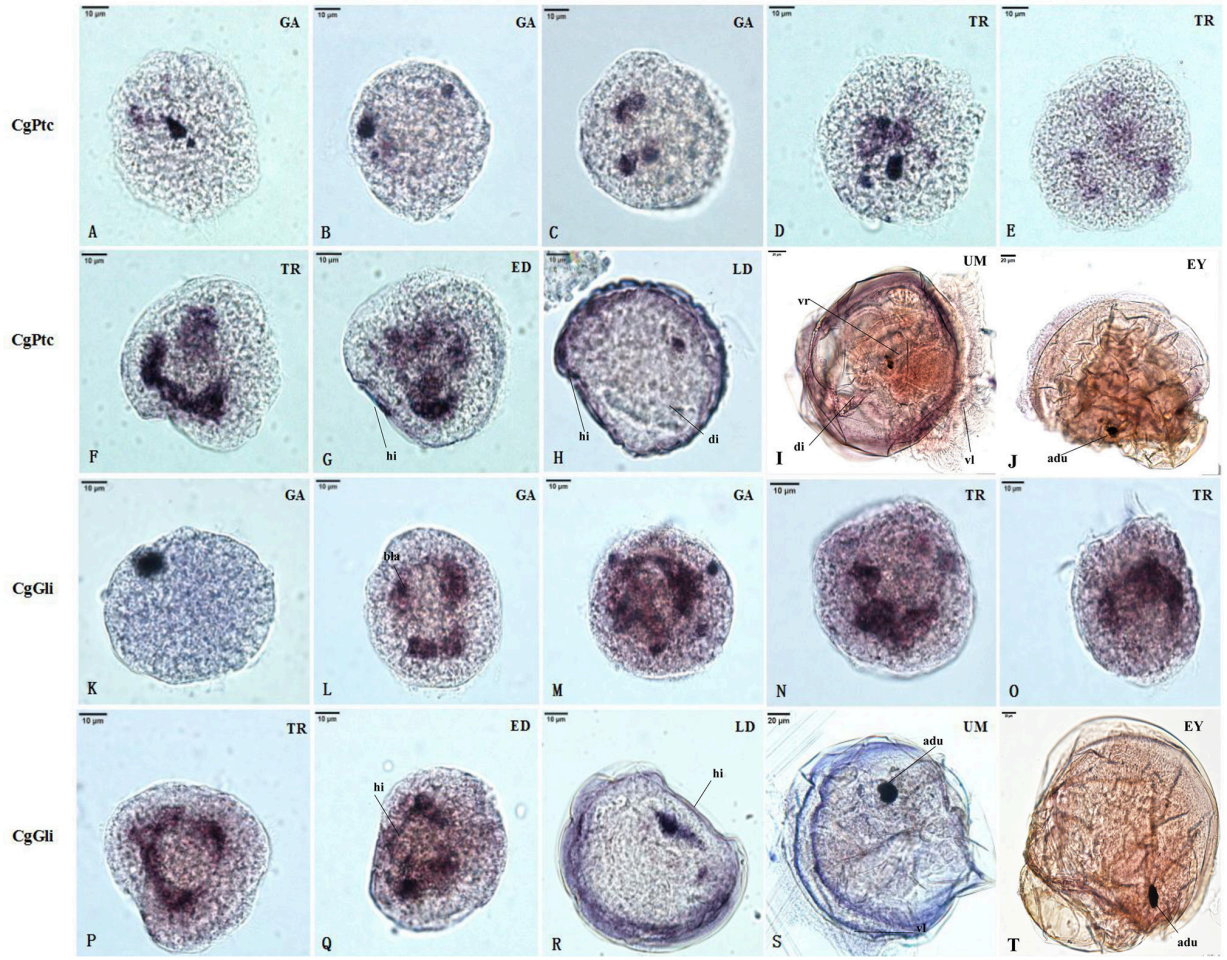
RNA localization patterns of key hedgehog pathway genes during embryonic to larval stage. **(A–C)** RNA localization patterns of CgPtc during gastrula stages. **(D–F)** RNA localization patterns of CgPtc during trochopore stages. **(G)** RNA localization pattern of CgPtc during the early D-shaped stage. **(H)** RNA localization pattern of CgPtc during the late D-shaped stage. **(I)** RNA localization patterns of CgPtc during umbo stages. **(J)** RNA localization patterns of CgPtc during eyed stages. **(K–M)** RNA localization patterns of CgGli during gastrula stages. **(N–P)** RNA localization patterns of CgGli during trochopore stages. **(Q)** RNA localization pattern of CgGli during the early D-shaped stage. **(R)** RNA localization pattern of CgGli during the late D-shaped stage. **(S)** RNA localization patterns of CgGli during umbo stages. **(T)** RNA localization patterns of CgGli during eyed stages. GA, gastrulae stage; TR, trochophore stage; ED, early D-shaped stage; LD, late D-shaped stage; UM, umbo larvae stage; EY, eyed-larvae stage; bla, blastopore; hi, hinge; di, digest gland; vl, vlum; um, umbo; fo, foot; adu, adductor.

The RNA localization patterns of key hedgehog pathway genes from fertilized eggs to eyed larvae was studied using whole-mount *in situ* hybridization (Figures 7, 8). The CgHh RNA localization pattern in early gastrula stage was a stained cell clusters at the middle of the embryo. The one cell clusters have an increase in number and size during gastrula stages. Several cell clusters were distributed randomly in the body of larvae in trochopore stage. The specific signal was observed in the velum position during umbo and eyed larvae stage. The RNA localization pattern of CgSmo was several stained clusters that were located symmetrically with respect to the midline of the larval body during gastrula stages. One cell cluster was observed from trochopore stage and D-shaped larvae stage. The CgSmo gene location was restricted to the gut and stomach domain in umbo stage. The specific staining was detectable at the foot in eyed stage. Similar to CgHh, the specific signaling of CgPtc was one cell cluster in early gastrula stage. The cell clusters were increased in embryos and larvae during gastrula and early trochopore stage larvae. The specific staining were clustered together and widespread during late trochopore stage and early D-shaped larvae. However, the specific signaling decreased in late D-shaped stage and located at the gut domain in the umbo larvae and adductor muscle domain in eyed larvae. The CgGli specific staining was one cell cluster in early gastrula stage and became more and more in late gastrula stage. The specific signaling was stronger and the distribution was widespread throughout the larval body during trochopore and early D-shaped larvae. One cell cluster was observed in adductor muscle domain in umbo and eyed larvae.

### Quantification of the Inhibitory Effect of Cyclopamine on the Expression of Key Hedgehog Pathway Genes

**Figure 9:**
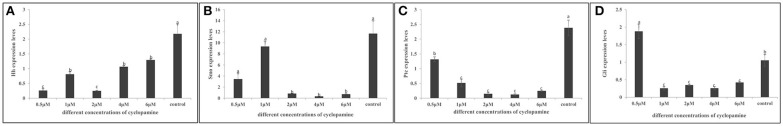
**(A–D)** Inhibitory effect of cyclopamine on the RNA Expression levels of key hedgehog pathway genes. The larvae in the figures was in the D-shaped stage. Different letters indicated significantly different (*P* < 0.05). The letters “a,” “b,” and “c” indicate the significance of the differences between two groups. Firstly, the whole mean of gene expression is ranked form large to small. The maximum average is marked the letter “a” and this average is compared with the following averages. Where the difference is not significant, the letter “a” is marked; otherwise, the letter “b” is marked. Secondly, the maximum average marked “b” is used as the standard deduction and compared with the following unmarked averages. Where the difference is not significant, the letter “b” is marked; otherwise, the letter “c” is marked. ad, adductor muscle; vr, velum retractor muscle; lr, larval retractor.

To evaluate if the expression of key hedgehog pathway genes was blocked by cyclopamine, we quantified the expression by means of RT- qPCR (Figure 9). We found that the expression of key genes were severely down-regulated in cyclopamine treated embryos. The effective inhibition concentration of CgHh and CgPtc was 0.5 μM. The expression of CgSmo and CgGli had a significant reduction when the embryos were treated with cyoclopamine at 2 and 1μM, respectively (*P* < 0.05). Results also showed that the expression level of CgPtc decreased with the increase of cyclopamine concentration.

### The Hedgehog Pathway Was Required for Normal Myogenesis in *C. gigas*

**Figure 10:**
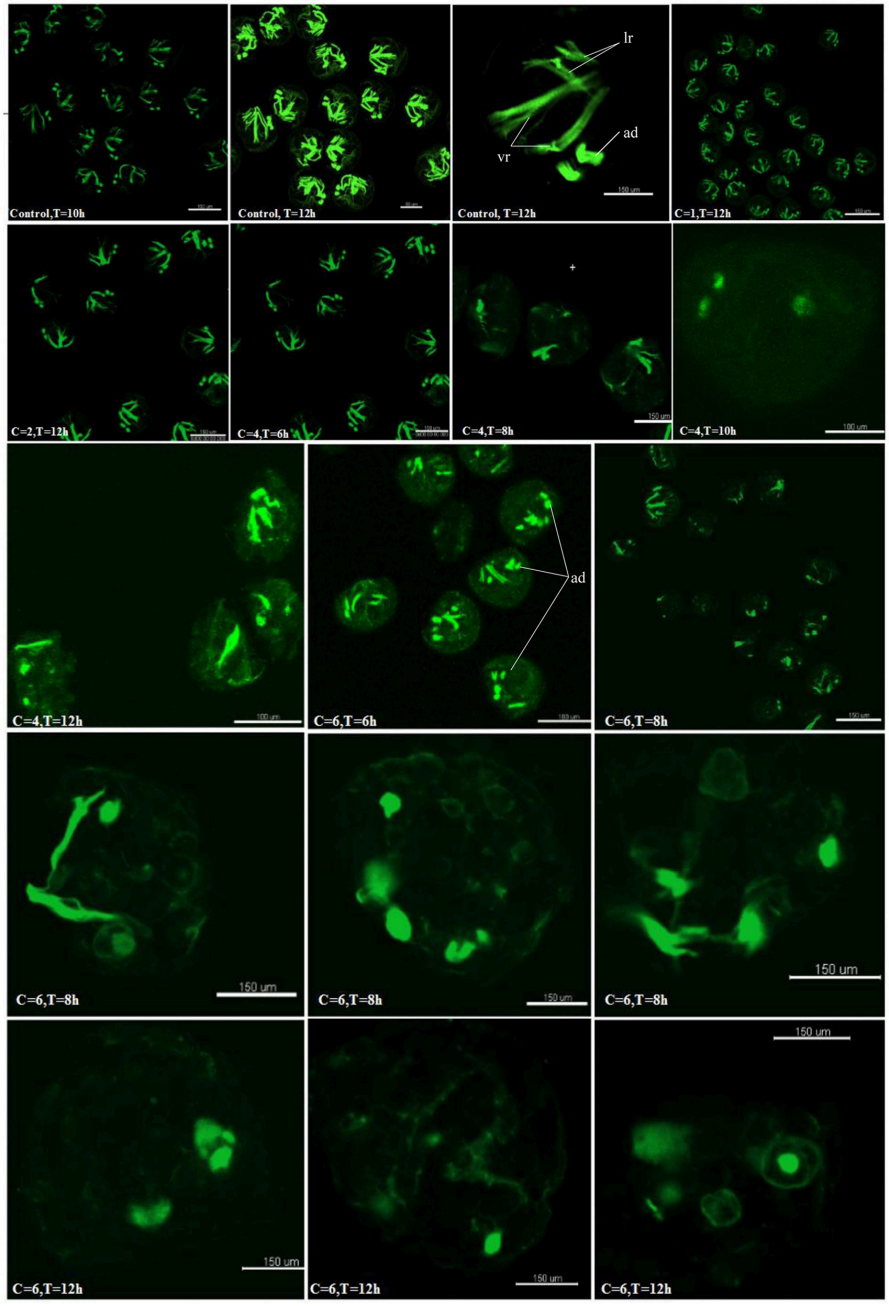
Myogenesis of cyclopamine treated larvae. C represent the concentration of the cyclopamine (μM), T represent the treated time (hour).

To examine if the hedgehog pathway was involved in normal muscle development in *C.gigas*, we investigated the myogenesis progress of cyclopamine treated larvae by fluorescence labeling of filamentous F-actin with Phalloidin-iFluor™ 488 Conjugate. Here, treatment with cyclopamine at 0.5, 1 and 2 μM for 12 h had no damage effect on muscle development and the muscle system of treatment groups was same to control groups which owned three pairs velum retractors, anterior adductor muscle and posterior adductor (Figure 10). The velum retractors and adductor muscles of larvae treated with cyclopamine at 4 μM for 6–12 h was significantly destroyed. The damage on larval muscle was mild after 6 h treatment and was severe after 12 h treatment. The damage effect on larval muscle was observed until the larvae were treated for 6 h at 6 μM and the larval velum retractor was destroyed severely. When the larvae were treated by cyclopamine at 6 μM for 8 h, the larval velum retractor was hardly presented. The damaged effect on larvae musculature in 12 h treatment was stronger than that in 6 h and the major musculature including velum retractor and adductor muscle of larvae was vanished.

## Discussion

### The Sequence Characteristics of Key Hedgehog Pathway Genes

The complete cDNA sequence and characterization of the hedgehog pathway genes in *C.gigas* were analyzed in the present study. The N-terminal of Hh was responsible for signaling, and the C-terminal autoprocessing Hint domain was in charge of adding cholesterol moiety to the N-terminal signaling domain (Gallet et al., 2003; Bijlsma et al., 2004). C-terminal to the Hint domain is a sterol recognition region [SSR; Thomas et al., 2008]. The CgHh owned conserved functional motifs including the N-terminal HH-signal domain and the C-terminal autoprocessing Hint domain. Moreover, a conserved intein N-terminal splicing motif were also detected in the Hint-N domain. These findings revealed that the CgHh possessed the primary structural and functional domains as present in typical Hh family. The high sequence similarity suggested the conserved function of Hh gene among Mollusca. In addition, similar to most invertebrates, there was only one Hh gene in *C.gigas*. In general, the obvious difference between invertebrates and vertebrates is that the key hedgehog pathway genes have been duplicated in vertebrates (Huangfu and Anderson, 2006). For example, there are five Hh homologs in zebrafish and three Hh homologs in the mouse respectively (Ekker et al., 1995; Bitgood et al., 1996; Zhang et al., 2001). However, there are only one Hh gene in most invertebrates such as *Drosophila* (Huangfu and Anderson, 2006), sea urchin (Warner et al., 2013), *Sepia officinalis* (Grimaldi et al., 2008). As an important receptor of Hh in hedgehog pathway, Ptc turns off the downstream signaling pathway in the absence of Hh ligand (Huangfu and Anderson, 2006). Based on the deduced amino acid sequence of CgPtc, we predicted that it contained a sterol-sensing domain (SSD) and patch domain, which were hallmarks of Ptc family members (Huangfu and Anderson, 2006). In addition, CgPtc owned 12 transmembrane domains which showed a high similarity among Mollusca (>70%). Apparently, this 12 transmembrane domains (TM) is common within the family of vertebrates and invertebrates including mouse (Goodrich et al., 1996), chicken (Marigo et al., 1996), *Drosophila* (Huangfu and Anderson, 2006), and *Sepia officinalis* (Grimaldi et al., 2008). Moreover, the phosphorylation sites of CgPtc was highly conserved among molluscan species (>70%). There was only one Ptc gene in *C.gigas* and it was consistent with the phenomenon that the key hedgehog pathway genes have been duplicated in vertebrates compared to invertebrates (Huangfu and Anderson, 2006). For example, both mice and zebrafish have two ptc homologs, while *Ciona* and *Drosophila* only oppose one Ptc gene (Goodrich et al., 1997; Wolff et al., 2003; Huangfu and Anderson, 2006). Smo is a membrane protein owning a seven TM domain structure (Alcedo et al., 1996). In *C. gigas*, CgSmo contained most common features of Smo including the 7-TM domain structure, FRI/ Frizzled conserved functional domains, Fruzzled (FZ) and G-protein coupled receptors family motifs. Multiple sequence alignment of Smo among invertebrates showed that the TM region was relatively conserved among species, while significant difference exists in the C-terminal domain (CTD). It was also found in *Drosophila* and experiments in the fly have demonstrated that the CTD is important for Smo activity (Jia et al., 2003; Nakano et al., 2004). In addition, similar to *Drosophila* we found that the predicted phosphorylated sites of CgSmo were mainly located in the C-terminal tail. Though the CTD of Smo was discrepant, the phosphorylation sites by protein kinase A (PKA) and casein kinase I (CKI) was conserved among invertebrates (Denef et al., 2000; Zhang et al., 2004). Because Smo phosphorylation coordinates Hh activation (Denef et al., 2000), we speculated that the function on Hh activation of Smo was conserved among invertebrates. Gli is a member of zinc-finger transcription, which has been identified as a downstream mediator of the hedgehog signaling pathway (Yang et al., 1997). According to the deduced amino acid sequence, CgGli owned most common structures including five conserved ZnF_C2H2 functional domains and zinc finger DNA binding domains. These results suggested that Gli was a relatively conserved genes among invertebrates.

### Temporal Expression Patterns of Key Hedgehog Pathway Genes

Quantitative analysis of the key genes in embryo-larval stages indicated that CgHh, CgPtc, CgGli mRNA began to accumulate during the blastula to gastrulation stages and accumulated throughout trochophore and into the D-shaped stage. Similar results were also found in the previous studies on sea urchin (Walton et al., 2006). In conclusion, the key genes mRNA expression levels were observed higher in the gastrulation and trochopore stags which were an early stage of muscle development. It suggests that hedgehog pathway was mainly involved in early muscle development such as the differentiation of mesodermal muscle. The key genes were expressed in all tested tissues, indicating that the hedgehog pathway participated in various physiological activities in *C.gigas*. The highest expression level of CgHh and CgPtc were observed in adductor smooth muscle, suggesting that the hedgehog pathway was connected with muscle development in *C. gigas*. Similar phenomenon also occurred in sea urchin (Warner et al., 2013).

### RNA Localization Patterns of Key Hedgehog Pathway Genes

The RNA localization patterns of CgHh and CgSmo were similar during gastrula and trochopore stages. In these stages, the irregular cell clusters were distributed randomly at the body of larvae. The specific staining of CgPtc and CgGli became stronger compared to CgHh and CgSmo during gastrula and trochopore stages. Moreover, the specific signal of CgPtc and CgGli were observed in velum retractors during the early D-shaped stages. The location patterns were accordance with myosin essential light chain (MELC) which was one muscle marker in *C. gigas* (Yu et al., 2017). These results suggested that CgPtc and CgGli were concerned with larval myogenesis in *C.gigas*. Similar data was also found in mouse (Wolff et al., 2003), *Sepia officinalis* (Grimaldi et al., 2008). The key pathway genes except CgSmo had a common localization that was near the hinge position during late D-shaped stage. The specific signals were observed in the velum, gut, stomach, adductor domain during umbo, and eyed larvae stage, suggesting that the signaling pathway might be involved in these organ development in *C. gigas*. Similarly, hedgehog signaling pathway have been confirmed to participate in muscle and gut development in chicken (Sukegawa et al., 2000), Amphioxus (Shimeld, 1999), zebrafish (Strähle et al., 1996), leech (Kang et al., 2003).

### Inhibitory Effect of Cyclopamine on the RNA Expression Levels of Key Hedgehog Pathway Genes

Cyclopamine, an antagonist of the hedgehog signaling, inhibits the pathway activation by binding directly to Smo (Chen et al., 2002). Hence, cyclopamine was often used to investigate the function of hedgehog pathway in various species including chicken (Incardona et al., 1998), Zebrafish (Wolff et al., 2003), *Sepia officinalis* (Grimaldi et al., 2008). In the study, we inhibited the hedgehog pathway with cyclopamine treatment in *C. gigas* larvae. The RNA expression levels of four key genes were severely down-regulated in treated larvae compared with normal larvae. Similarly, the RNA expression of Ptc and Hh was reduced significantly in cyclopamine- treated embryos in chicken (Incardona et al., 1998). Same phenomenon was also found in Zebrafish and *Sepia officinalis* that the expression levels of Ptc was down-regulated in treatment groups (Wolff et al., 2003; Grimaldi et al., 2008). In addition, the effective inhibition concentration was different among species. In present study, we suggested that the effective inhibition concentration of CgHh and CgPtc was 0.5 μM. The effective concentration of CgSmo was 2 μM. The expression level of Ptc in zebrafish and *Sepia officinalis* was reduced significantly when the drug concentration was 15 and 10 μM respectively (Wolff et al., 2003; Grimaldi et al., 2008). We speculated the discrepancy on effective inhibition concentration of cyclopamine might be relevant to some reasons including the size of embryos, the treated stage or treated time. Our results also found that there was only the expression level of CgPtc decreased with the increase of cyclopamine concentration. Though the expression levels of CgHh, CgSmo and CgGli were down-regulated in treatment groups, there was no a consistent trend as the concentration of cyclopamine increases. We speculated that this situation might be due to the fact that the larvae used for analysis are mixed sample.

### A Conserved Role for Hedgehog Signaling in Muscle Development

To verify if the hedgehog pathway was involved in normal muscle development in *C. gigas*, we investigated the changes in larval muscle phenotype by fluorescence labeling of F-actin. The data showed that as the treatment concentration and time increased, the degree of damage on larval muscle was more serious. Similar phenomenon was also found in zerbrafish (Wolff et al., 2003). Moreover, we suggested that hedgehog signaling pathway had a conserved role in muscle development among species. In sea urchin embryo, hedgehog signaling was vital for gut muscle development (Walton et al., 2006). In cuttlefish, the hedgehog pathway was involved in the formation of striated muscle fibers (Grimaldi et al., 2008). Hedgehog signaling was also proved to promote the proliferation and differentiation of muscle cells in zebrafish (Feng et al., 2006).

## Conclusion

The present study suggested that hedgehog signaling pathway was a conserved pathway in sequence characteristic, expression profile and physiological function among invertebrates. For physiological function, we thought hedgehog pathway was involved in myogenesis of *C.gigas*. Firstly, the key genes of hedgehog pathway had a relatively high expression level in gastrulation or trochophore stage when myogenesis was just beginning to develop. Next, cyclopamine blocked the pathway and lead to RNA expression levels of CgHh, CgPtc and CgSmo were severely down-regulated. The velum retractors and adductor muscles of larvae treated with cyclopamine were significantly destroyed. In conclusion, we thought that hedgehog signaling pathway participated in myogenesis of *C.gigas*, but detailed regulatory mechanism of the signaling on muscle development is not yet clear and remains to explore.

## Author contributions

HL performed the experiments, analyzed the data, drafted the manuscript, and generated all figures. HY designed the study, supervised the research, contributed to data interpretation, and writing of the manuscript. QL supervised the project and contributed to data interpretation and writing of the manuscript. All authors provided input and read and approved the final version of the manuscript.

### Conflict of interest statement

The authors declare that the research was conducted in the absence of any commercial or financial relationships that could be construed as a potential conflict of interest.

## References

[B1] Alcedo J. Ayzenzon M. Von Ohlen T. Noll M. Hooper J. E. (1996). The Drosophila smoothened gene encodes a seven-pass membrane protein, a putative receptor for the hedgehog signal. Cell 86, 221–232. 10.1016/S0092-8674(00)80094-X 8706127

[B2] Bijlsma M. F. Spek C. A. Peppelenbosch M. P. (2004). Hedgehog: an unusual signal transducer. Bioessays 26, 387–394. 10.1002/bies.20007 15057936

[B3] Bitgood M. J. Shen L. McMahon A. P. (1996). Sertoli cell signaling by Desert hedgehog regulates the male germline. Curr. Biol. 6, 298–304. 10.1016/S0960-9822(02)00480-3 8805249

[B4] Blagden C. S. Currie P. D. Ingham P. W. Hughes S. M. (1997). Notochord induction of zebrafish slow muscle mediated by Sonic hedgehog. Genes Dev. 11, 2163–2175. 10.1101/gad.11.17.2163 9303533 PMC275397

[B5] Braun T. Rudnicki M. A. Arnold H. H. Jaenisch R. (1992). Targeted inactivation of the muscle regulatory gene Myf-5 results in abnormal rib development and perinatal death. Cell 71, 369–382. 10.1016/0092-8674(92)90507-9 1423602

[B6] Chen J. K. Taipale J. Cooper M. K. Beachy P. A. (2002). Inhibition of Hedgehog signaling by direct binding of cyclopamine to smoothened. Genes Dev. 16, 2743–2748. 10.1101/gad.1025302 12414725 PMC187469

[B7] Cornell R. A. Eisen J. S. (2005). Notch in the pathway: the roles of Notch signaling in neural crest development. Semin. Cell Dev. Biol. 16, 663–672. 10.1016/j.semcdb.2005.06.009 16054851

[B8] Currie P. D. Ingham P. W. (1996). Induction of a specific muscle cell type by a hedgehog-like protein in zebrafish. Nature 382, 452–55. 10.1038/382452a0 8684485

[B9] Denef N. Neubuser D. Perez L. Cohen S. M. (2000). Hedgehog induces opposite changes in turnover and subcellular localization of patched and smoothened. Cell 102, 521–531. 10.1016/S0092-8674(00)00056-8 10966113

[B10] Du S. J. Devoto S. H. Westerfield M. Moon R. T. (1997). Positive and negative regulation of muscle cell identity by members of the hedgehog and TGF-beta gene families. J. Cell Biol. 139, 145–156. 10.1083/jcb.139.1.145 9314535 PMC2139815

[B11] Du Y. Zhang L. Xu F. Huang B. Zhang G. Li L. (2013). Validation of housekeeping genes as internal controls for studying gene expression during Pacific oyster (Crassostrea gigas) development by quantitative real-time PCR. Fish. Shellfish. Immunol. 24, 939–945. 10.1016/j.fsi.2012.12.007 23357023

[B12] Duprez D. Fournier-Thibault C. Le Douarin N. (1998). Sonic hedgehog induces proliferation of committed skeletal muscle cells in the chick limb. Development 125, 495–505. 9425144 10.1242/dev.125.3.495

[B13] Echelard Y. Epstein D. J. St-Jacques B. Shen L. Mohler J. McMahon J.. (1993). Sonic hedgehog, a member of a family of putative signaling molecules, is implicated in the regulation of CNS polarity. Cell 75, 1417–1430. 10.1016/0092-8674(93)90627-3 7916661

[B14] Ekker S. C. Ungar A. R. Greenstein P. von Kessler D. P. Porter J. A. Moon R. T.. (1995). Patterning activities of vertebrate hedgehog proteins in the developing eye and brain. Curr. Biol. 5, 944–955. 10.1016/S0960-9822(95)00185-0 7583153

[B15] Feng X. Adiarte E. G. Devoto S. H. (2006). Hedgehog acts directly on the zebrafish dermomyotome to promote myogenic differentiation. Dev. Biol. 300, 736–746. 10.1016/j.ydbio.2006.08.056 17046741

[B16] Gallet A. Rodriguez R. Ruel L. Therond P. P. (2003). Cholesterol modification of hedgehog is required for trafficking and movement, revealing an asymmetric cellular response to hedgehog. Dev. Cell 4, 191–204. 10.1016/S1534-5807(03)00031-5 12586063

[B17] Goodrich L. V. Johnson R. L. Milenkovic L. McMahon J. A. Scott M. P. (1996). Conservation of the hedgehog/patched signaling pathway from flies to mice: induction of a mouse patched gene by hedgehog. Genes Dev. 10, 301–312. 10.1101/gad.10.3.301 8595881

[B18] Goodrich L. V. Milenkovic L. Higgins K. M. Scott M. P. (1997). Altered neural cell fates and medulloblastoma in mouse patched mutants. Science 277, 1109–1113. 10.1126/science.277.5329.1109 9262482

[B19] Goulding M. Lumsden A. Paquette A. J. (1994). Regulation of Pax-3 expression in the dermomyotome and its role in muscle development. Development 120, 957–971. 7600971 10.1242/dev.120.4.957

[B20] Grimaldi A. Tettamanti G. Acquati F. (2008). A hedgehog homolog is involved in muscle formation and organization of *Sepia officinalis* (mollusca) mantle. Dev. Dyn. 237, 659–671. 10.1002/dvdy.21453 18265019

[B21] Heretsch P. Tzagkaroulaki L. Giannis A. (2010). Modulators of the hedgehog signaling pathway. Bioorg. Med. Chem. 18, 6613–6624. 10.1016/j.bmc.2010.07.038 20708941

[B22] Huangfu D. Anderson K. V. (2006). Signaling from Smo to Ci/Gli: conservation and divergence of hedgehog pathways from Drosophila to vertebrates. Development 133, 3–14. 10.1242/dev.02169 16339192

[B23] Incardona J. P. Gaffield W. Kapur R. P. (1998). The teratogenic Veratrum alkaloid cyclopamine inhibits sonic hedgehog signal transduction. Development 125, 3553–3562. 9716521 10.1242/dev.125.18.3553

[B24] Incardona J. P. Gaffield W. Lange Y. Cooney A. Pentchev P. G. Liu S. (2000). Cyclopamine inhibition of Sonic hedgehog signal transduction is not mediated through effects on cholesterol transport. Dev. Biol. 224, 440–452. 10.1006/dbio.2000.9775 10926779

[B25] Ingham P. W. McMahon A. P. (2001). Hedgehog signaling in animal development: paradigms and principles. Genes Dev. 15, 3059–3087. 10.1101/gad.938601 11731473

[B26] Ingham P. W. Placzek M. (2006). Orchestrating ontogenesis: variations on a theme by sonic hedgehog. Nat. Rev. Genet. 7, 841–850. 10.1038/nrg1969 17047684

[B27] Jia J. Amanai K. Wang G. Tang J. Wang B. Jiang J. (2002). Shaggy/GSK3 antagonizes hedgehog signalling by regulating cubitus interruptus. Nature 416, 548–552. 10.1038/nature733 11912487

[B28] Jia J. Tong C. Jiang J. (2003). Smoothened transduces hedgehog signal by physically interacting with Costal2/fused complex through its C-terminal tail. Genes Dev. 17, 2709–2720. 10.1101/gad.1136603 14597665 PMC280620

[B29] Kang D. Huang F. Li D. Shankland M. Gaffield W. Weisblat D. A. (2003). A hedgehog homolog regulates gut formation in leech Helobdella. Development 130, 1645–57. 10.1242/dev.00395 12620988

[B30] Kirilly D. Spana E. P. Perrimon N. Padgett R. W. Xie T. (2005). BMP signaling is required for controlling somatic stem cell self-renewal in the Drosophila ovary. Dev. Cell 9, 651–662. 10.1016/j.devcel.2005.09.013 16256740

[B31] Kruger M. Mennerich D. Fees S. Schafer R. Mundlos S. Braun T. (2001). Sonic hedgehog is a survival factor for hypaxial muscles during mouse development. Development 128, 743–752. 11171399 10.1242/dev.128.5.743

[B32] Kumar S. Stecher G. Tamura K. (2016). MEGA7: molecular evolutionary genetics analysis version 7.0 for bigger datasets. Mol. Biol. Evol. 33, 1870–1874. 10.1093/molbev/msw054 27004904 PMC8210823

[B33] Lee C. S. Buttitta L. Fan C. M. (2001). Evidence that the WNT-inducible growth arrest-specific gene 1 encodes an antagonist of sonic hedgehog signaling in the somite. Proc. Natl. Acad. Sci. U.S.A. 98, 11347–11352. 10.1073/pnas.201418298 11572986 PMC58732

[B34] Livak K. J. Schmittgen T. D. (2001). Analysis of relative gene expression data using realtime quantitative PCR and the 2(-Delta Delta C (T)) method. Methods 25, 402–408. 10.1006/meth.2001.1262 11846609

[B35] Marigo V. Scott M. P. Johnson R. L. (1996). Conservation in hedgehog signaling: induction of a chicken patched homolog by Sonic hedgehog in the developing limb. Development 122, 1225–1233. 8620849 10.1242/dev.122.4.1225

[B36] Matus D. Q. Magie C. R. Pang K. (2008). The Hedgehog gene family of the cnidarian, Nematostella vectensis, and implications for understanding metazoan hedgehog pathway evolution. Dev. Biol. 313, 501–518. 10.1016/j.ydbio.2007.09.032 18068698 PMC2288667

[B37] Méthot N. Basler K. (2000). Suppressor of fused opposes hedgehog signal transduction by impeding nuclear accumulation of the activator form of Cubitus interruptus. Development 127, 4001–4010. 10952898 10.1242/dev.127.18.4001

[B38] Nakano Y. Nystedt S. Shivdasani A. A. Strutt H. Thomas C. Ingham P. W. (2004). Functional domains and sub-cellular distribution of the hedgehog transducing protein smoothened in Drosophila. Mech. Dev. 121, 507–518. 10.1016/j.mod.2004.04.015 15172682

[B39] Nusse R. (2003). Wnts and hedgehogs: lipid-modified proteins and similarities in signaling mechanisms at the cell surface. Development 130, 5297–5305. 10.1242/dev.00821 14530294

[B40] Nüsslein-Volhard C. Wieschaus E. (1980). Mutations affecting segment number and polarity in Drosophila. Nature 287, 795–801. 10.1038/287795a0 6776413

[B41] Østerlund T. Kogerman P. (2006). Hedgehog signalling: how to get from Smo to Ci and Gli. Trends. Cell. Biol. 16, 176–180. 10.1016/j.tcb.2006.02.004 16516476

[B42] Pérez-Bercoff Å. Koch J. Bürglin T. R. (2005). LogoBar: bar graph visualization of protein logos with gaps. Bioinformatics 22, 112–114. 10.1093/bioinformatics/bti761 16269415

[B43] Price M. A. Kalderon D. (2002). Proteolysis of the hedgehog signaling effector cubitus interruptus requires phosphorylation by glycogen synthase kinase 3 and casein kinase 1. Cell 108, 823–835. 10.1016/S0092-8674(02)00664-5 11955435

[B44] Riddle R. D. Johnson R. L. Laufer E. Tabin C. (1993). Sonic hedgehog mediates the polarizing activity of the ZPA. Cell 75, 1401–1416. 10.1016/0092-8674(93)90626-2 8269518

[B45] Robert X. Gouet P. (2014). Deciphering key features in protein structures with the new ENDscript server. Nucl. Acids Res. 42, 320–324. 10.1093/nar/gku316 24753421 PMC4086106

[B46] Roelink H. Augsburger A. Heemskerk J. Korzh V. Norlin S. Ruiz I.. (1994). Floor plate and motor neuron induction by vhh-1, a vertebrate homolog of hedgehog expressed by the notochord. Cell 76, 761–775. 10.1016/0092-8674(94)90514-2 8124714

[B47] Shimeld S. M. (1999). The evolution of the hedgehog gene family in chordates: insights from amphioxus hedgehog. Dev. Genes Evol. 209, 40–47. 10.1007/s004270050225 9914417

[B48] Strähle U. Blader P. Ingham P. W. (1996). Expression of axial and sonic hedgehog in wildtype and midline defective zebrafish embryos. Int. J. Dev. Biol. 40, 929–940. 8946241

[B49] Sukegawa A. Narita T. Kameda T. Saitoh K. Nohno T. Iba H.. (2000). The concentric structure of the developing gut is regulated by Sonic hedgehog derived from endodermal epithelium. Development 127, 1971–1980. 10751185 10.1242/dev.127.9.1971

[B50] Thisse C. Thisse B. (2008). High resolution *in situ* hybridization on whole-mount zebrafish embryo. Nat. Protoc. 3, 59–69. 10.1038/nprot.2007.514 18193022

[B51] Thomas N. A. Koudijs M. van Eeden F. J. M. Joyner A. L. Yelon D. (2008). Hedgehog signaling plays a cell-autonomous role in maximizing cardiac developmental potential. Development 135, 3789–3799. 10.1242/dev.024083 18842815 PMC4213142

[B52] Villavicencio E. H. Walterhouse D. O. Iannaccone P. M. (2000). The sonic hedgehog–patched-gli pathway in human development and disease. Am. J. Hum. Genet. 67, 1047–1054. 10.1016/S0002-9297(07)62934-6 11001584 PMC1288546

[B53] Walton K. D. Croce J. C. Glenn T. D. Croce J. C. Glenn T. D. Wu S. Y.. (2006). Genomics and expression profiles of the Hedgehog and Notch signaling pathways in sea urchin development. Dev. Biol. 300, 153–164. 10.1016/j.ydbio.2006.08.064 17067570 PMC1880897

[B54] Wang Q. Li Q. Kong L. Yu R. (2012). Response to selection for fast growth in the second generation of Pacific oyster (*Crassostrea gigas*). J. Ocean Univ. China 11, 413–418. 10.1007/s11802-012-1909-7

[B55] Warner J. F. McCarthy A. M. Morris R. L. (2013). Hedgehog signaling requires motile cilia in the sea urchin. Mol. Biol. Evol. 31, 18–22. 10.1093/molbev/mst176 24124205 PMC3879447

[B56] Weed M. Mundlos S. Olsen B. R. (1997). The role of sonic hedgehog in vertebrate development. Matrix Biol. 16, 53–58. 10.1016/S0945-053X(97)90072-X 9205942

[B57] Wolff C. Roy S. Ingham P. W. (2003). Multiple muscle cell identities induced by distinct levels and timing of hedgehog activity in the zebrafish embryo. Curr. Biol. 13, 1169–81. 10.1016/S0960-9822(03)00461-5 12867027

[B58] Yang J. T. Liu C. Z. Villavicencio E. H. (1997). Expression of human GLI in mice results in failure to thrive, early death, and patchy Hirschsprung-like gastrointestinal dilatation. Mol. Med. 3:826. 10.1007/BF03401719 9440116 PMC2230283

[B59] Yu H. Li H. Li Q. (2017). Molecular characterization and expression profiles of myosin essential light chain gene in the Pacific oyster Crassostrea gigas. Comp. Biochem. Physiol. B Biochem. Mol. Biol. 213, 1–7. 10.1016/j.cbpb.2017.07.007 28735975

[B60] Zhang C. Williams E. H. Guo Y. Lum L. Beachy P. A. (2004). Extensive phosphorylation of smoothened in hedgehog pathway activation. Proc. Natl. Acad. Sci. U.S.A. 101, 17900–17907. 10.1073/pnas.0408093101 15598741 PMC535705

[B61] Zhang X. M. Ramalho-Santos M. McMahon A. P. (2001). Smoothened mutants reveal redundant roles for Shh and Ihh signaling including regulation of L/R symmetry by the mouse node. Cell 106, 781–92. 10.1016/S0092-8674(01)00385-3 11517919

[B62] Zhu Q. Zhang L. Li L. Que H. Zhang G. F. (2016). Expression characterization of stress genes under high and low temperature stresses in the Pacific oyster, *Crassostrea gigas*. Mar. Biotechnol. 18, 176–188. 10.1007/s10126-015-9678-0 26746430

