# Supplementary figures and images for: Molecular Characterization of the Hedgehog Signaling Pathway and Its Necessary Function on Larval Myogenesis in the Pacific Oyster Crassostrea gigas
Source: Front Physiol. 2018 Dec 5;9:1536. doi: 10.3389/fphys.2018.01536 (PMC6290081; doi:10.3389/fphys.2018.01536)

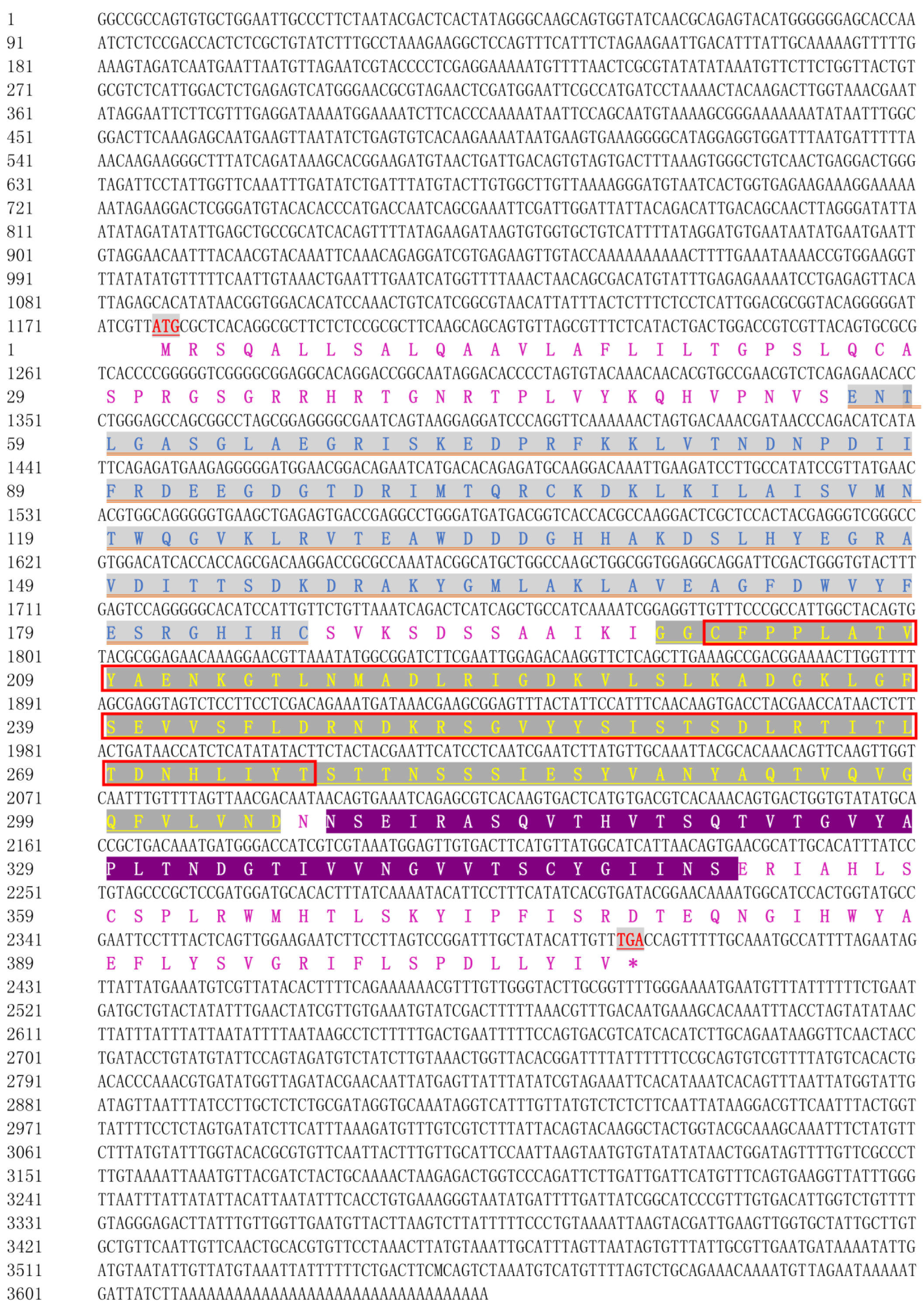

Supplement: Supplementary file 1 [file Image_1.TIF]

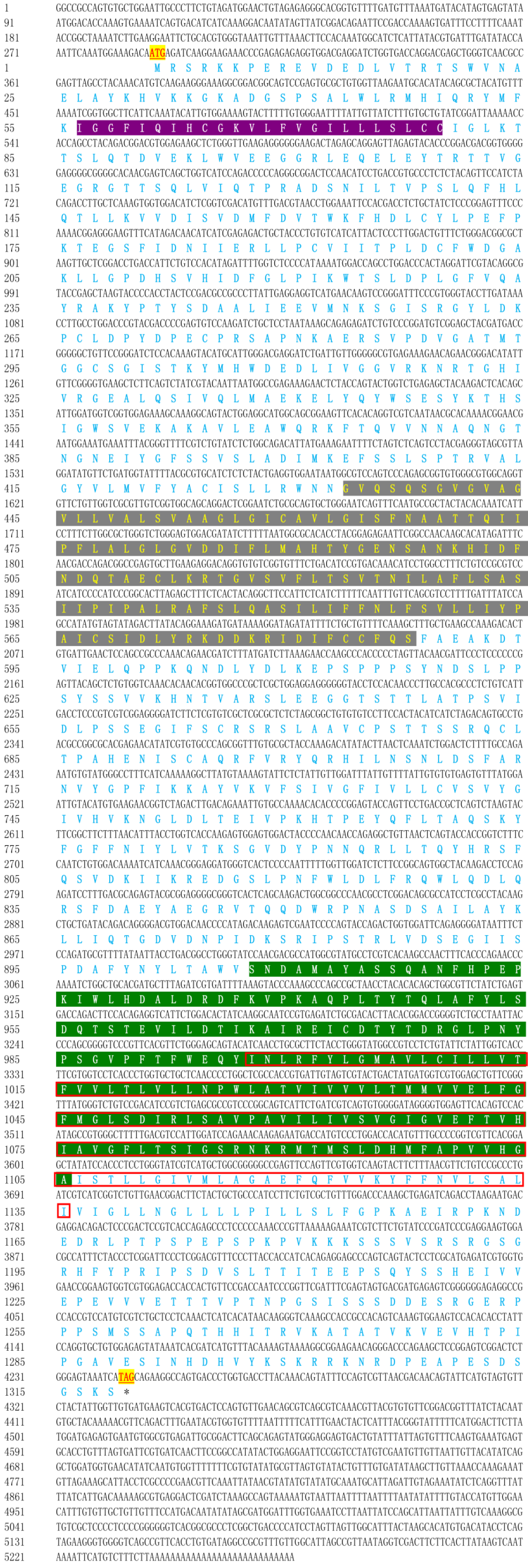

Supplement: Supplementary file 2 [file Image_2.TIF]

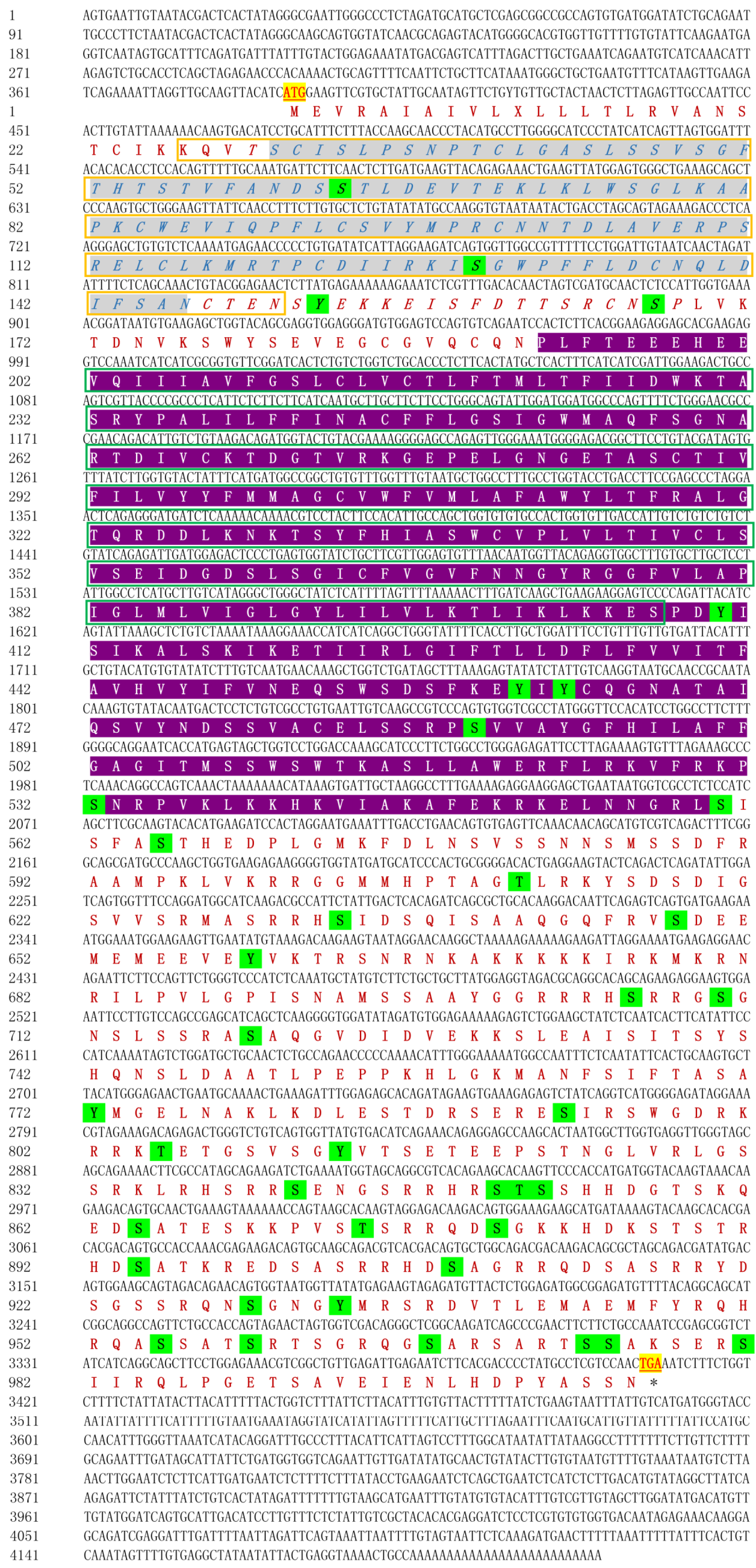

Supplement: Supplementary file 3 [file Image_3.TIF]

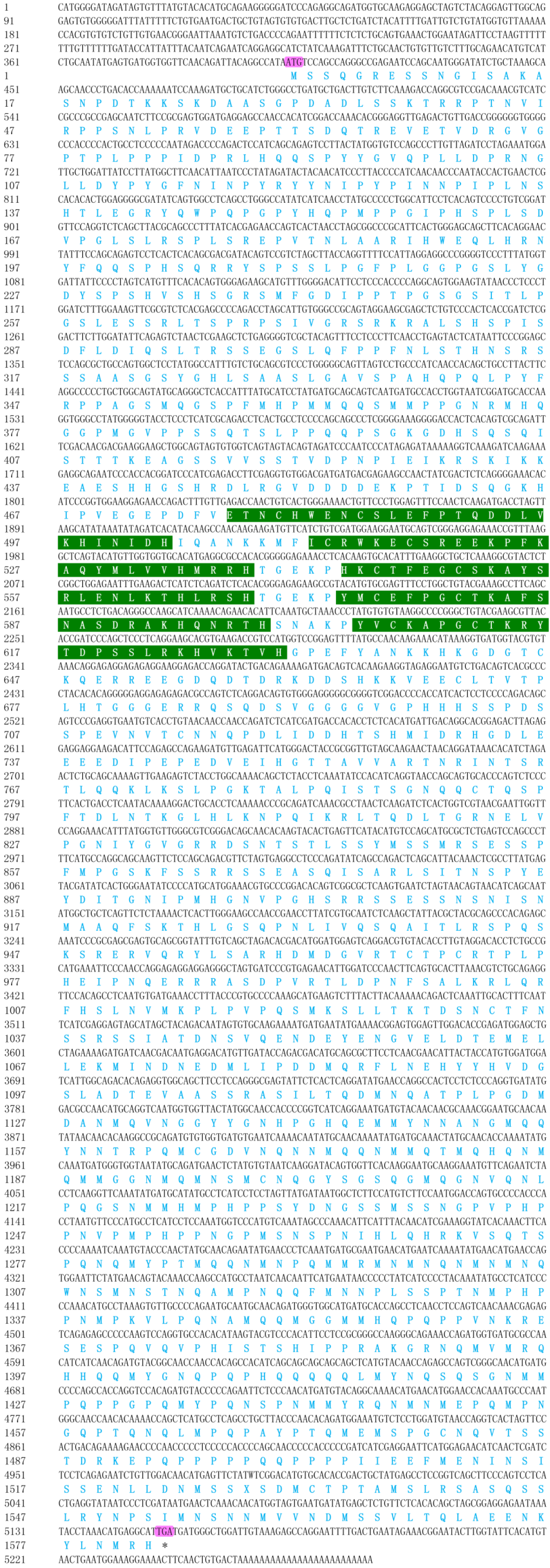

Supplement: Supplementary file 4 [file Image_4.TIF]

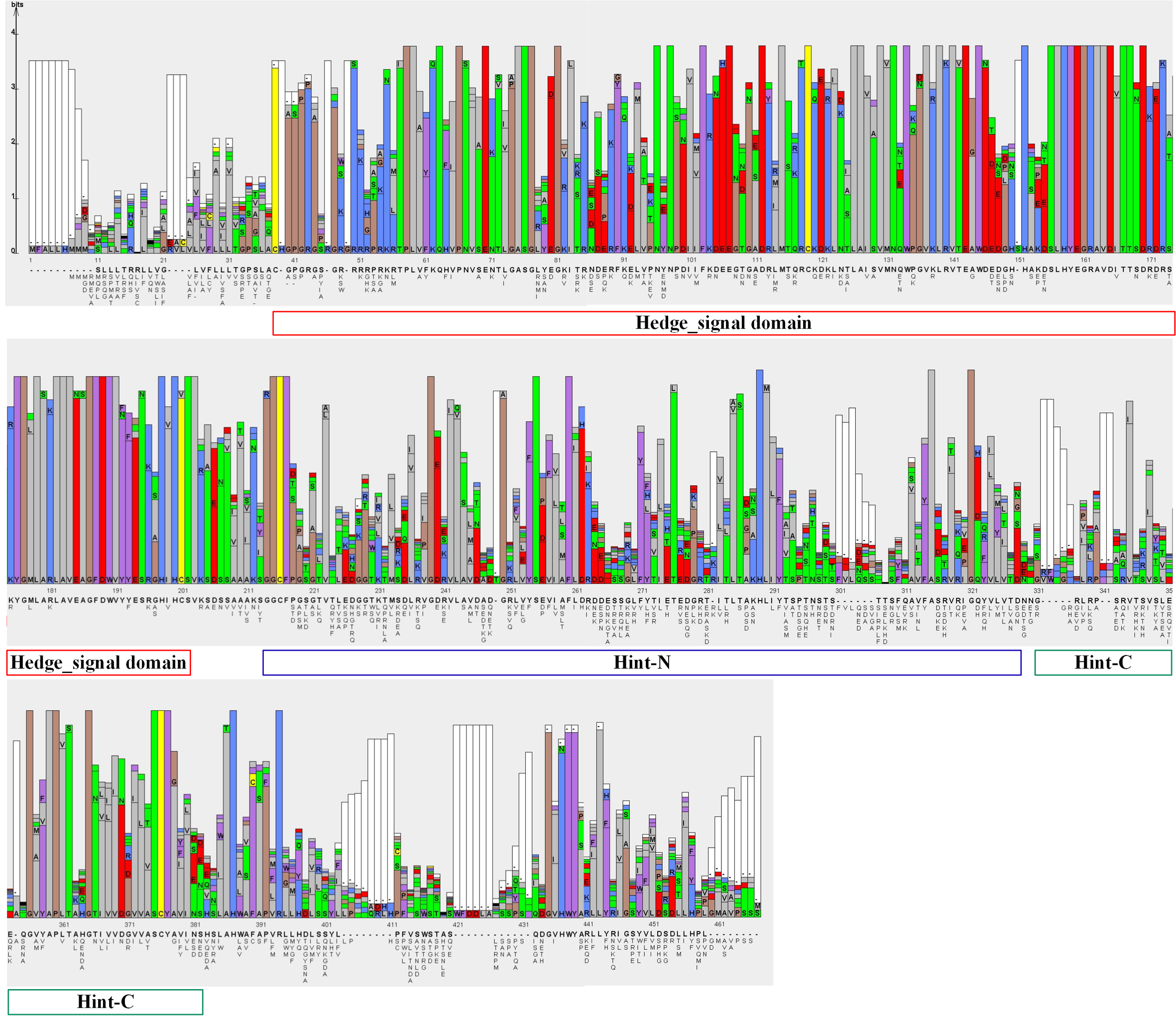

Supplement: Supplementary file 5 [file Image_5.TIF]

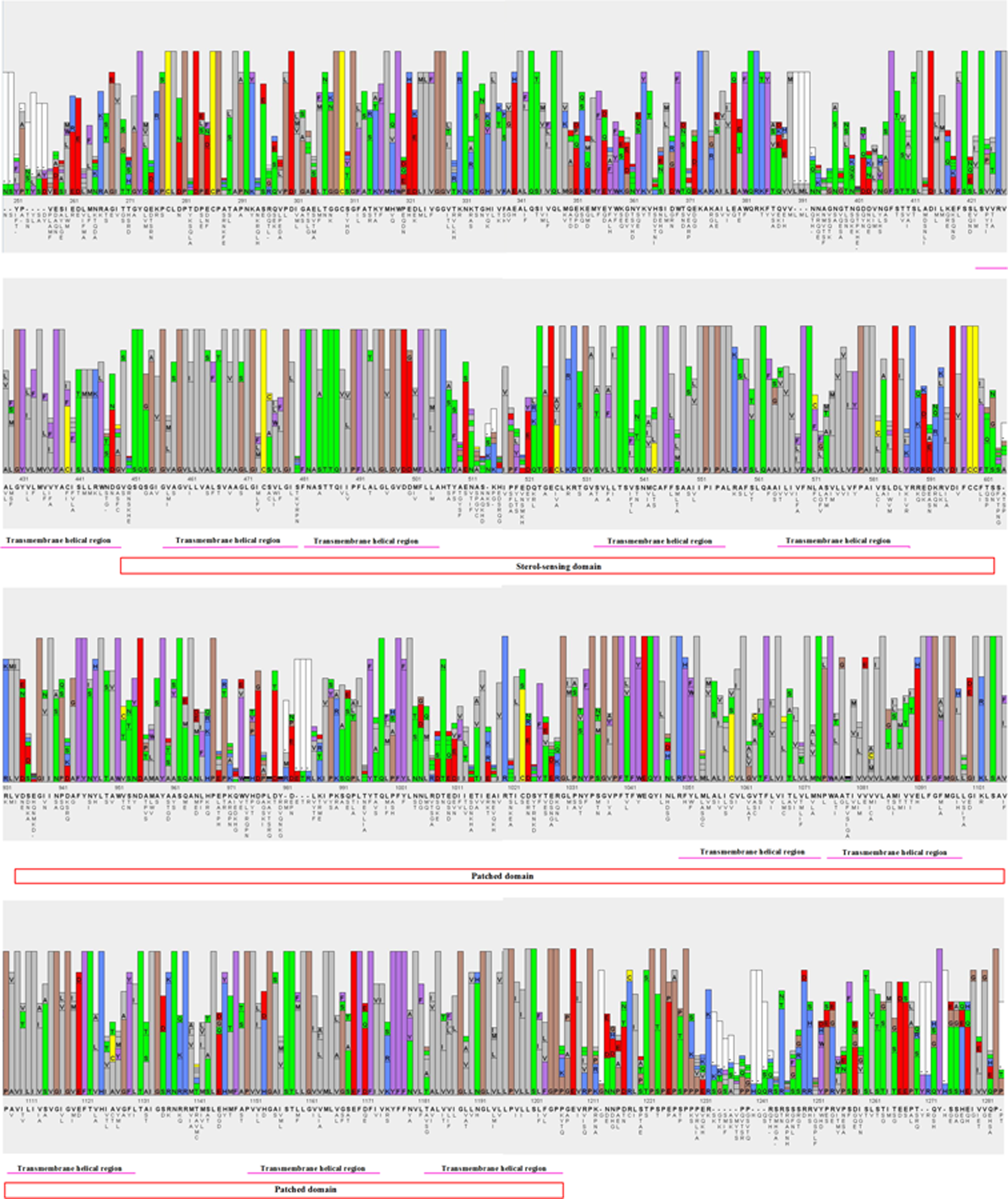

Supplement: Supplementary file 6 [file Image_6.TIF]

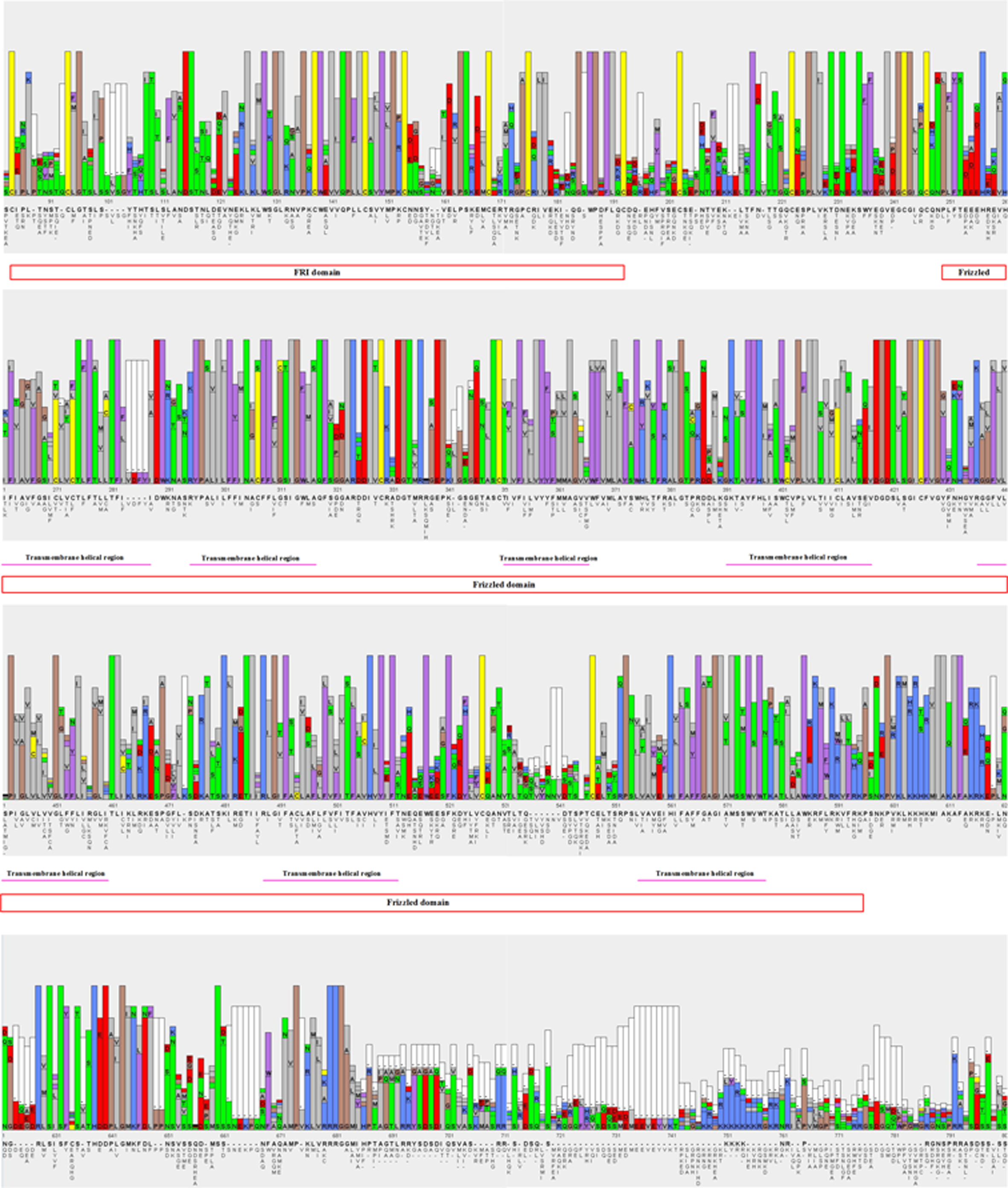

Supplement: Supplementary file 7 [file Image_7.TIF]

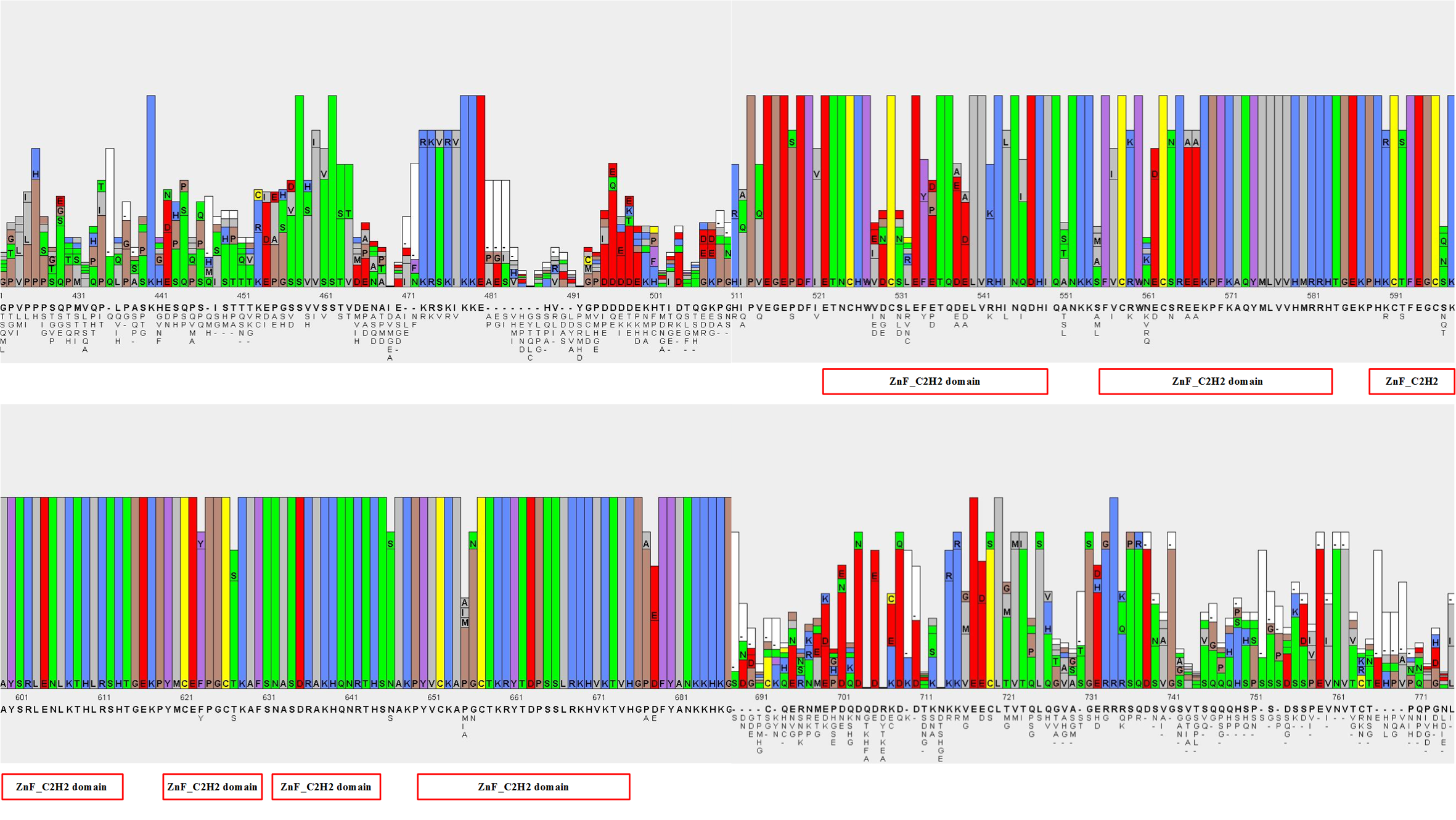

Supplement: Supplementary file 8 [file Image_8.TIF]
