# Supplementary material for: Molecular Characterization of the Hedgehog Signaling Pathway and Its Necessary Function on Larval Myogenesis in the Pacific Oyster Crassostrea gigas
Source: Front Physiol. 2018 Dec 5;9:1536. doi: 10.3389/fphys.2018.01536 (PMC6290081; doi:10.3389/fphys.2018.01536)
Supplement: Supplementary file 9 [file Data_Sheet_1.DOCX]

Supplementary Material

**Molecular characterization of the hedgehog signaling pathway and its necessary function on larval myogenesis in the Pacific oyster *Crassostrea gigas***

Huijuan Li^1^, Qi Li^1,2, *^, Hong Yu^1,2^

^1^ *Key Laboratory of Mariculture, Ministry of Education, Ocean University of China, Qingdao 266003, China*

^2^ *Laboratory for Marine Fisheries Science and Food Production Processes, Qingdao National Laboratory for Marine Science and Technology*

** Correspondence:*

*Prof. Qi Li*

*qili66@ouc.edu.cn*

**Figure legends**

S1. Nucleotide and deduced amino acid sequences of CgHh. The start (ATG) and the termination (TGA) are underlined in red bold. The conserved functional domain including HH-signal, Hint-N and Hint-C are highlighted in light grey, charcoal grey and dark purple. A intein N-terminal splicing motif are indicated with red box.

S2. Nucleotide and deduced amino acid sequences of CgPtc. A transmembrane helix region is highlighted in purple. The [Sterol-sensing](http://pfam.xfam.org/family?id=Sterol-sensing) and Patched conservative functional structure domain are highlighted in grey and green. The 7 TM receptor with intracellular HD hydrolase domain is marked in red box

S3. Nucleotide and deduced amino acid sequences of CgSmo. The FRI and Frizzled conservative functional domains are highlighted in grey and purple. The Fruzzled (FZ) and G-protein coupled receptors family motifs are marked in yellow and dark green box respectively. The phosphorylation sites are highlighted in light green.

S4. Nucleotide and deduced amino acid sequences of CgGli. The start (ATG) and the termination (TGA) are highlighted in red. Five conservative ZnF_C2H2 functional domains are highlighted in green.

S5. Protein logo generated from multiple sequence alignment of the CgHhamino acid sequence. Under the logo is a consensus sequence under which residues found at a position are listed in decreasing order of frequency. The conservative functional structure domains are marked in color box.

S6. Protein logo generated from multiple sequence alignment of the CgPtc amino acid sequence. Under the logo is a consensus sequence under which residues found at a position are listed in decreasing order of frequency. The conservative functional structure domains are marked in color box. The transmembrane helical domains are underlined in red.

S7. Protein logo generated from multiple sequence alignment of the CgSmo amino acid sequence. Under the logo is a consensus sequence under which residues found at a position are listed in decreasing order of frequency. The conservative functional structure domains are marked in red box. The transmembrane helical domains are underlined in red.

S8. Protein logo generated from multiple sequence alignment of the CgGli amino acid sequence. Under the logo is a consensus sequence under which residues found at a position are listed in decreasing order of frequency. The conservative functional structure domains are marked in red box.
